# Chromosome Missegregation Triggers Tumor Cell Pyroptosis and Enhances Anti‐Tumor Immunotherapy in Colorectal Cancer

**DOI:** 10.1002/advs.202409769

**Published:** 2025-02-04

**Authors:** Wei Duan, Rendy Hosea, Lingxian Wang, Cao Ruan, Fuqiang Zhao, Jingyi Liu, Hezhao Zhao, Makoto Miyagishi, Shourong Wu, Vivi Kasim

**Affiliations:** ^1^ Key Laboratory of Biorheological Science and Technology Ministry of Education College of Bioengineering Chongqing University Chongqing 400044 China; ^2^ The 111 Project Laboratory of Biomechanics and Tissue Repair College of Bioengineering Chongqing University Chongqing 400044 China; ^3^ Department of Gastrointestinal Surgery Chongqing University Cancer Hospital Chongqing University Chongqing 400030 China; ^4^ Life Science Innovation School of Integrative and Global Majors University of Tsukuba Tsukuba Ibaraki 305‐0006 Japan; ^5^ Chongqing Key Laboratory of Translational Research for Cancer Metastasis and Individualized Treatment Chongqing University Cancer Hospital Chongqing University Chongqing 400030 China

**Keywords:** anti‐tumor immunotherapy, CD8^+^ T cell, chromosome missegregation, cytosolic dsDNA response, micronucleus, pyroptosis, yin yang 2

## Abstract

Immune checkpoint inhibitor (ICI) therapy is a promising anti‐tumor therapeutic strategy; however, its efficacy in solid tumors is limited. Chromosome missegregation is common in various solid tumors; however, its role in tumor progression remains poorly understood, and its correlation with ICI is yet to be explored. Here, it is found that increased chromosome missegregation promotes tumor immune microenvironment, and eventually immunotherapeutic efficacy, by triggering pyroptosis. yin yang 2 (YY2) is identified as a mitotic checkpoint regulator, which promotes chromosome missegregation by upregulating *BUB1B* transcription. Increased chromosome missegregation promoted the formation of micronuclei and release of double‐stranded DNA (dsDNA) into the cytosol, triggering an AIM2‐mediated cytosolic dsDNA response. The subsequent pyroptosis strengthened the tumor immune microenvironment, thereby enhancing immunoinfiltration and cytotoxicity of CD8^+^ T cells, while preventing their exhaustion. Finally, through in vitro and in vivo experiments, it is demonstrated that combining *YY2* overexpression‐induced chromosome missegregation/cytosolic dsDNA response and PD‐1 inhibitor significantly enhanced the efficacy of ICI immunotherapy in microsatellite instable and microsatellite stable colorectal cancer cells. Together, these findings provide new insights on the role of chromosome missegregation in triggering cytosolic dsDNA response‐mediated pyroptosis and modulating the tumor immune microenvironment, suggesting a novel strategy for improving ICI therapeutic efficacy in colorectal cancer.

## Graphical abstract

Immune system disorders and chromosome missegregation are two of the major hallmarks of tumor. The relationship between tumor immune and chromosome missegregation is still unclear. This study reveals that YY2 high expression‐mediated chromosome missegregation/micronuclei formation triggers pyroptosis and CTL activation by activating AIM2/caspase‐1/GSDMD cytosolic dsDNA response, and YY2/anti‐PD‐L1 combinatorial anti‐tumor therapeutic strategy.

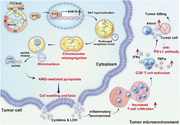

## 1 Introduction

Immune checkpoint inhibitor (ICI) therapies, such as anti‐cytotoxic T‐lymphocyte associated protein‐4, anti‐programmed cell death‐1 (anti‐PD‐1), and anti‐programmed death ligand‐1 (anti‐PD‐L1), activate the innate immune system and improve T cell anti‐tumor activity.^[^ ^1^, ^2^ ^]^ ICI therapies significantly ameliorate patient survival and, therefore, have emerged as promising anti‐tumor strategies. However, their efficacy in treating solid tumors is greatly hindered by antigen loss, T cell dysfunction, insufficient infiltration, tumor immunosuppression, and deregulated tumor immunometabolism.^[^ ^3^, ^4^ ^]^ Therefore, increasing the sensitivity of tumor cells, especially in solid tumors, to ICI therapies could expand their clinical application.

Chromosomal instability (CIN), a hallmark of cancer found in various solid tumors,^[^ ^5^, ^6^ ^]^ refers to the high rate of chromosome gain and loss caused by mitotic errors.^[^ ^7^, ^8^ ^]^ Chromosome missegregation, which is observed in 60%–80% of human tumors,^[^ ^9^ ^]^ is associated with a faulty mitotic process and is one of the major causes of CIN.^[^ ^10^ ^]^ Certain level of chromosome missegregation, which leads to a certain increase of CIN level, is a driving force of tumorigenesis, as it increases tumor heterogeneity and adaptability, thereby facilitating tumor cell proliferation, evolution, metastasis, and drug resistance.^[^ ^11^, ^12^ ^]^ However, exacerbating chromosome missegregation can aggravate mitotic collapse, triggering the formation of chromosome bridges and micronuclei, further enhancing CIN.^[^ ^13^, ^14^ ^]^ Eventually, this results in genetic catastrophe and cell death, which halts tumorigenesis.^[^ ^15^, ^16^ ^]^ Therefore, as proposed by the “just‐right” model of CIN, maintaining a balance between genomic chaos and heterogeneity by restricting chromosome missegregation rates within an optimal range is crucial for tumor cell survival.^[^ ^13^, ^17^ ^]^ Chromosome missegregation can lead to an increased tumor mutational burden and disrupt normal biological processes, which might result in more neoantigen formation and immune cells recognition.^[^ ^18^ ^]^ However, the correlation between chromosome missegregation with tumor immunity, along with whether it could improve the efficacy of ICI therapy in solid tumors are yet to be explored; furthermore, the mechanism underlying chromosome missegregation is still poorly understood.

In this study, we aimed to investigate the mechanism regulating chromosome missegregation in tumor cells. We unprecedently reveal its role in tumor immunity and its effect on the efficacy of anti‐tumor immunotherapy. Using cross‐omics analysis, as well as cell and animal models, we show that yin yang 2 (YY2) is a mitotic checkpoint regulator, which facilitates chromosome missegregation by promoting *budding uninhibited by benzimidazoles 1 homolog beta* (*BUB1B*) transcription. This subsequently triggers absent in melanoma 2 (AIM2)/caspase‐1/gasdermin D (GSDMD)‐mediated pyroptosis, thereby enhancing tumor immunity and the efficacy of anti‐PD‐L1 immunotherapy in both microsatellite instable (MSI) and microsatellite stable (MSS) tumor cells. These findings suggest a novel combinatorial strategy, based on promoting chromosome missegregation, to increase the efficacy of ICI therapies against solid tumors.

## 2 Results

### 2.1 YY2 Triggers Pyroptosis by Inducing Chromosome Missegregation

**Figure 1:**
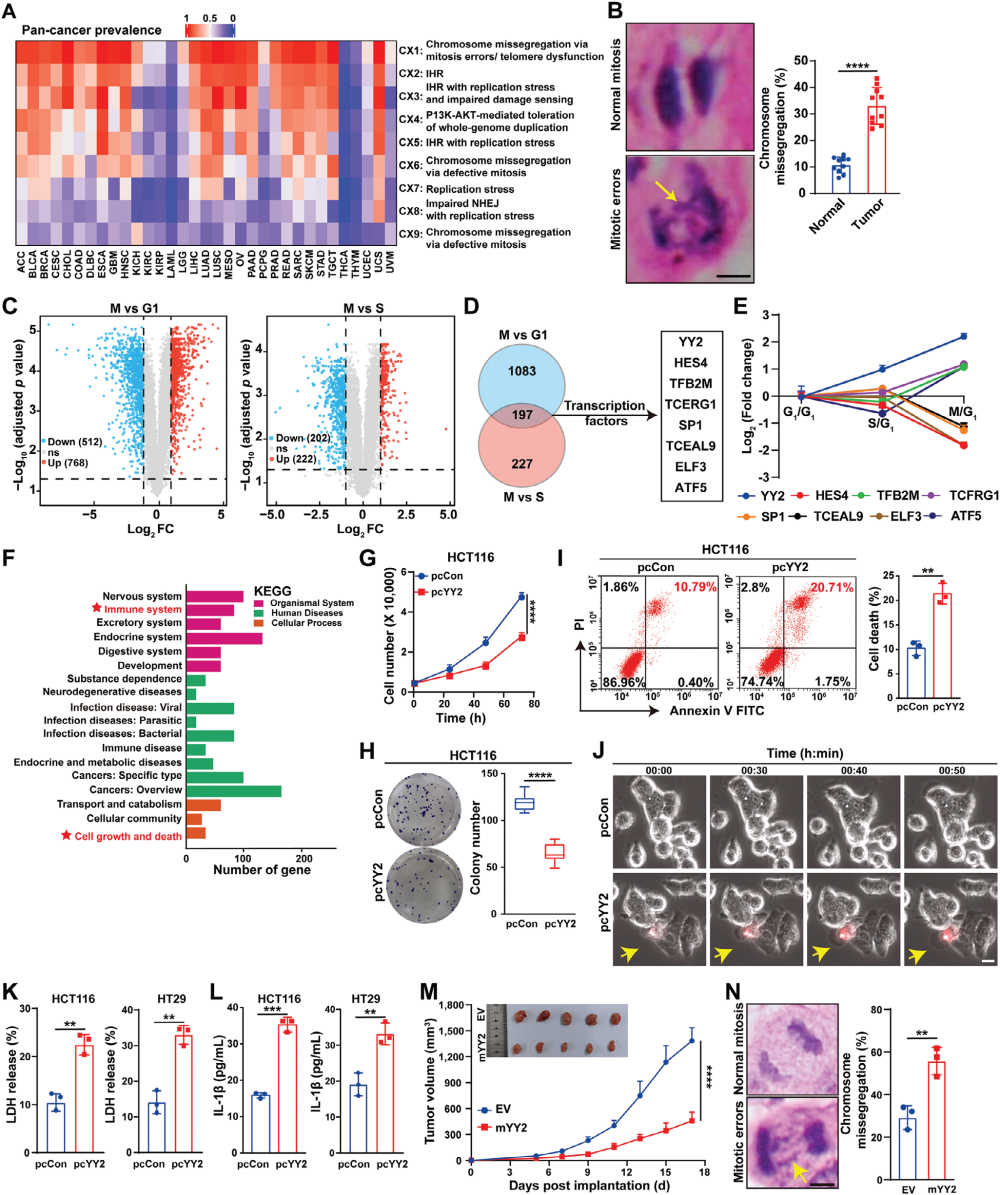
*YY2* overexpression triggers pyroptosis by inducing chromosome missegregation. A) Putative causes of CIN in different tumor types from TCGA datasets (CX1, CX6, and CX9: mitotic signature, CX2, CX3, and CX5: impaired homologous recombination signature, CX4: whole‐genome duplication signature, CX7: replication stress signature, CX8: impaired non‐homologous end joining signature). B) Percentage of cells with chromosome missegregation in clinical CRC samples. Representative images (indicated by arrow, scale bars: 5 µm) and quantification results are shown (each dot represents one slide, total cell counted > 100 mitotic cells/group). C) Volcano plot of DEGs in different cell cycle phases of synchronized HCT116 cells obtained from RNA‐seq (|fold‐change| > 2‐fold; *p* value < 0.05). D,E) Mitotic phase specific transcription factors as obtained by cross‐sectioning DEGs in M phase versus G_1_ phase and M phase versus S phase (D; |fold‐change| > 2‐fold, *p* value < 0.05), and their expression levels in each cycle phase (E) as obtained from RNA‐seq results. F) KEGG enrichment analysis of DEGs in *YY2*‐overexpressing HCT116 cells obtained from RNA‐seq. G–I) Viability at indicated time‐points (G), colony formation potential (H), and cell death rate (I) (Annexin V^+^/PI^+^ cells) of *YY2*‐overexpressing HCT116 cells. J) Cell death of *YY2*‐overexpressing HCT116 cells, as examined using PI staining and time‐lapse microscopy (arrows: PI‐positive cells; scale bars: 100 µm). K,L) LDH (K) and IL‐1β (L) released from *YY2*‐overexpressing CRC cells. M) Tumor volume and morphological images of syngeneic graft tumors formed by *YY2*‐overexpressing MC38 cells (*n* = 5/group). N) Percentage of cells with chromosome missegregation in the syngeneic graft tumor lesions formed by *YY2*‐overexpressing MC38 cells. Representative image of chromosome missegregation (indicated by arrow, scale bars: 5 µm) and quantification results are shown (each dot represents one slide, total cell counted >100 mitotic cells/group). Cells transfected with pcCon were used as control. Quantification data are shown as mean ± S.D. All data were obtained from three independent experiments, unless otherwise indicated. pcCon: pcEF9‐Puro; EV: empty lentivirus; ns: not significant; **p < 0.01; ***p < 0.001; ****p < 0.0001.

To comprehensively analyze the correlation between CIN and tumors, we screened The Cancer Genome Atlas, and found greater ploidy diversity, a marker of CIN,^[^ ^19^, ^20^ ^]^ in various tumors (Figure S1A, Supporting Information). Analysis of the pan‐cancer compendium of CIN signatures revealed that chromosome missegregation during mitosis might be one of the major causes of CIN in tumors (**Figure** **1**A). This was further confirmed by increased chromosome missegregation in clinical colorectal cancer (CRC) tissues, as indicated by the total ratio of cells with lagging chromosomes and chromosome bridges (Figure 1B).

Impaired M phase regulation is the main cause of chromosome missegregation. To identify novel M phase regulators, we synchronized cells in G_1_ using serum starvation, collected cells from different phases (G_1_, S, and M), and subjected them to RNA‐sequencing (RNA‐seq) (Figure S1B,C, Supporting Information). Notably, all cells used in this study have been confirmed as negative for mycoplasma contamination (Figure S1D, Supporting Information). RNA‐seq results were validated by analyzing the expression patterns of *CCNE2*, *PCNA*, and *AURKA*, which are specific for G_1_, S, and M phases, respectively (Figure S1E, Supporting Information). Analysis of differentially expressed genes (DEGs) with more than twofold change revealed 768 upregulated and 512 downregulated genes in M phase compared to the G_1_ phase (Figure 1C, left panel), along with 222 upregulated and 202 downregulated genes in M phase compared to the S phase (Figure 1C, right panel).

Despite their function in regulating gene expression, the role of transcription factors in the cell cycle remains poorly understood.^[^ ^21^ ^]^ Even though mutations in mitotic checkpoint genes are rare, alterations in their expression are common in tumors, indicating that transcriptional regulation might play a vital role in M phase.^[^ ^22^ ^]^ Thus, we cross‐analyzed DEGs in M phase compared to G_1_ or S phase. YY2, HES4, TFB2M, TCERG1, SP1, TCEAL9, ELF3, and ATF5 transcription factors were differentially expressed in M phase, with YY2 showing a gradual increase with cell cycle progression and the most significant difference (Figure 1D,E). Furthermore, YY2 expression peaked in M phase (Figure S1F, Supporting Information). This trend was confirmed with cells from each cell cycle phase synchronized using the double‐thymidine block (Figure S1G–I, Supporting Information).

YY2 is a multifunctional transcription factor which regulates stem cells activity and acts as a tumor suppressor.^[^ ^23^, ^24^, ^25^, ^26^ ^]^ Tissue microarrays revealed downregulation of YY2 in CRC (Figure S1J,K, Supporting Information) along with a positive correlation between YY2 expression and overall survival (Figure S1L, Supporting Information). *YY2* overexpression prolonged the mitotic time from 50.04 to 70.94 min (Figure S1M; Videos S1 and S2, Supporting Information), while also increasing the number of cells with excessive chromosome numbers and chromosome missegregation, as indicated by chromosome bridges and lagging chromosomes (Figure S1N,O, Supporting Information). Concomitantly, it promoted the formation of micronuclei, which are nucleus‐like structures in the cytosol comprising extra‐chromosomal double‐stranded DNA (dsDNA) that originated from a lagging chromosome and/or a chromosome bridge enclosed by a nuclear envelope‐like membrane (Figure S1P, Supporting Information). These data indicated that YY2 might be involved in the regulation of sister chromatid segregation.

Mitotic checkpoint is a surveillance mechanism in M phase that prevents premature sister chromatid segregation until all chromosomes are properly attached to the mitotic spindle.^[^ ^27^ ^]^ *YY2* overexpression increased the levels of securin and cyclin B, whose degradation triggered sister chromatid segregation and M phase progression, respectively, indicating that *YY2* overexpression leads to mitotic checkpoint hyperactivation (Figure S2A, Supporting Information). Treatment with reversine, an inhibitor of mitotic checkpoint, lowered YY2‐induced securin and cyclin B expression to control levels (Figure S2B, Supporting Information), along with a reversal of chromosome missegregation and micronuclei formation (Figure S2C,D, Supporting Information). These findings showed that YY2 regulates chromosome missegregation by inducing mitotic checkpoint hyperactivation.

Next, we investigated the role of YY2 in determining tumor cell fate. Kyoto Encyclopedia of Genes and Genomes (KEGG) analysis showed DEG enrichment in “cell growth and death” and “immune system” pathways in *YY2*‐overexpressing cells (Figure 1F).^[^ ^23^ ^]^ Validation using CRC cells revealed that *YY2* overexpression significantly decreased viability and colony formation potential (Figure 1G,H; Figure S2E,F, Supporting Information), while increasing cell death rates (Figure 1I; Figure S2G, Supporting Information).

Intriguingly, time‐lapse analysis revealed that *YY2* overexpression induced cell swelling and membrane rupture (Figure 1J; Videos S3 and S4, Supporting Information), which are typically associated with a unique form of immune‐induced cell death known as pyroptosis.^[^ ^28^ ^]^ Accordingly, we examined the effect of YY2 on the release of pyroptosis markers lactate dehydrogenase (LDH) and interleukin (IL)‐1β. *YY2* overexpression increased the release of LDH and IL‐1β from CRC cells (Figure 1K,L). Together with KEGG analysis, these results indicate that YY2 might trigger pyroptosis.

We next explored the correlation between YY2‐induced pyroptosis and chromosomal missegregation. Treatment with reversine suppressed the increase in LDH and IL‐1β release (Figure S2H,I, Supporting Information). It also canceled the negative effect of *YY2* overexpression on CRC cell viability (Figure S2J–M, Supporting Information). These results pointed to a possible correlation between *YY2*‐induced chromosome missegregation and pyroptosis.

We next performed syngeneic graft experiments using mouse CRC MC38 cells, which also showed decreased viability and increased cell death upon *YY2* overexpression (Figure S2N,O, Supporting Information). *YY2* overexpression robustly suppressed MC38 cell tumorigenic potential (Figure 1M), most plausibly by inducing pyroptosis in vivo, as evidenced by more IL‐1β and propidium iodide‐positive cells in syngeneic graft lesions (Figure S2P–R, Supporting Information). Furthermore, increased chromosome missegregation was observed in tumor lesions formed by *YY2*‐overexpressing MC38 cells (Figure 1N). Together, these results suggested that YY2 could trigger pyroptosis, which suppresses tumorigenic potential, as well as the potential correlation between chromosome missegregation and pyroptosis.

### 2.2 YY2‐Mediated Pyroptosis Enhances T Cell Proliferation and Activation

**Figure 2:**
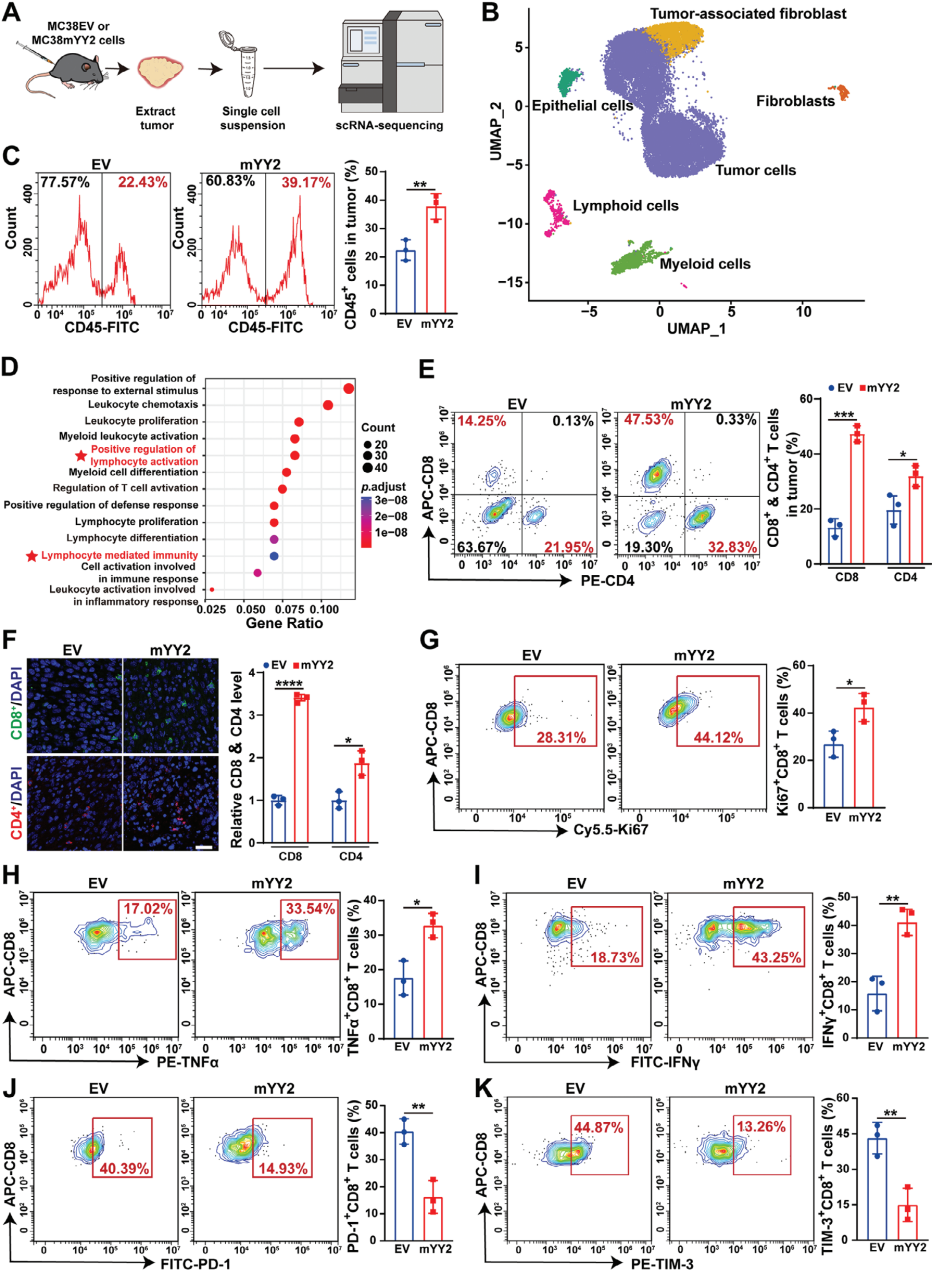
*YY2* overexpression enhances CD8^+^ T cell infiltration and activation. A) Schematic diagram showing scRNA‐seq procedures of syngeneic graft tumor formed by MC38 cells. B) Annotation of main cell populations identified by scRNA‐seq in syngeneic graft tumors formed by MC38 cells. The uniform manifold approximation and projection (UMAP) plot shows merged samples from both syngeneic graft tumor lesions formed by *YY2*‐overexpressing and empty lentivirus (EV)‐infected MC38 cells. C) Percentage of CD45^+^ cells in syngeneic graft tumor lesions formed by *YY2*‐overexpressing MC38 cells (three tumors from three mice for each group). D) GO enrichment analysis of upregulated genes in CD45^+^ cells in syngeneic graft tumor lesions formed by *YY2*‐overexpressing MC38 cells. E,F) Percentages of CD8^+^ and CD4^+^ T cells in syngeneic graft tumor lesions formed by *YY2*‐overexpressing MC38 cells as examined using flow cytometry (E; three tumors from three mice for each group) and immunofluorescent staining (F; left panels: representative images, scale bars: 200 µm; right panels: quantification results, each dot represents quantification results of two slides from the same mice, total 6 slides from 3 mice). G–K) Percentages of Ki67^+^CD8^+^ (G), TNFα^+^CD8^+^ (H), IFNγ^+^CD8^+^ (I), PD‐1^+^CD8^+^ (J), and TIM‐3^+^CD8^+^ (K) T cells in syngeneic graft tumor lesions formed by *YY2*‐overexpressing MC38 cells (three tumors from three mice for each group). Quantification data are shown as mean ± S.D. EV: empty lentivirus; *p < 0.05; **p < 0.01; ***p < 0.001; ****p < 0.0001.

The pyroptosis‐induced inflammatory environment promotes immune cell recruitment, proliferation, and activation, thereby enhancing anti‐tumor immunity and tumor suppressive effect.^[^ ^29^ ^]^ We performed single‐cell RNA‐sequencing (scRNA‐seq) using tumor lesions formed by *YY2*‐overexpressing MC38 and control cells to investigate the influence of YY2‐induced pyroptosis on the immune system (**Figure** **2**A). Besides tumor cells, cluster analysis identified epithelial cells, lymphoid cells, myeloid cells, fibroblasts, and tumor‐associated fibroblasts (Figure 2B). Increased infiltration of immune cells was confirmed by numerous CD45^+^ cells in the tumor lesions formed by *YY2*‐overexpressing cells (Figure 2C). Gene Ontology (GO) and KEGG analyses of CD45^+^ cells revealed enrichment for features associated with lymphocyte immune activation (Figure 2D; Figure S3A, Supporting Information). Further GO analysis using upregulated genes in CD45^+^CD11b^−^ cells, which represents lymphocytes, showed enrichment for T cells activation and proliferation (Figure S3B, Supporting Information). Similarly, KEGG analysis of CD45^+^CD3^+^ cells, which represents T cells, confirmed strong T cell activation and proliferation (Figure S3C, Supporting Information). These results pointed to the activation and proliferation of lymphocytes, especially T cells, in tumor lesions formed by *YY2*‐overexpressing cells.

T cells can be classified as CD8^+^ T cells (cytotoxic T cells, CTLs) and CD4^+^ T cells. While slightly enhancing the numbers of CD4^+^ T cells, *YY2* overexpression particularly increased that of CD8^+^ T cells (Figure 2E,F). Furthermore, it promoted CTL proliferation, as indicated by abundant Ki67^+^CD8^+^ T cells (Figure 2G), as well as the expression of tumor necrosis factor (TNF) receptor superfamily 18 (Tnfrsf18) and Tnfrsf9 (Figure S3D,E, Supporting Information), which are often upregulated when T cells are stimulated.^[^ ^30^ ^]^ Furthermore, we observed an increase in TNFα^+^CD8^+^ and interferon‐gamma (IFNγ)^+^CD8^+^ T cells (Figure 2H,I), suggesting enhanced anti‐tumor CTL activity, and a decrease in cells positive for T cell exhaustion markers PD‐1 and TIM‐3 (Figure 2J,K). These results implied that YY2 promotes proliferation and activation of CTLs, and mitigates their exhaustion while stimulating their tumor‐killing potential.

**Figure 3:**
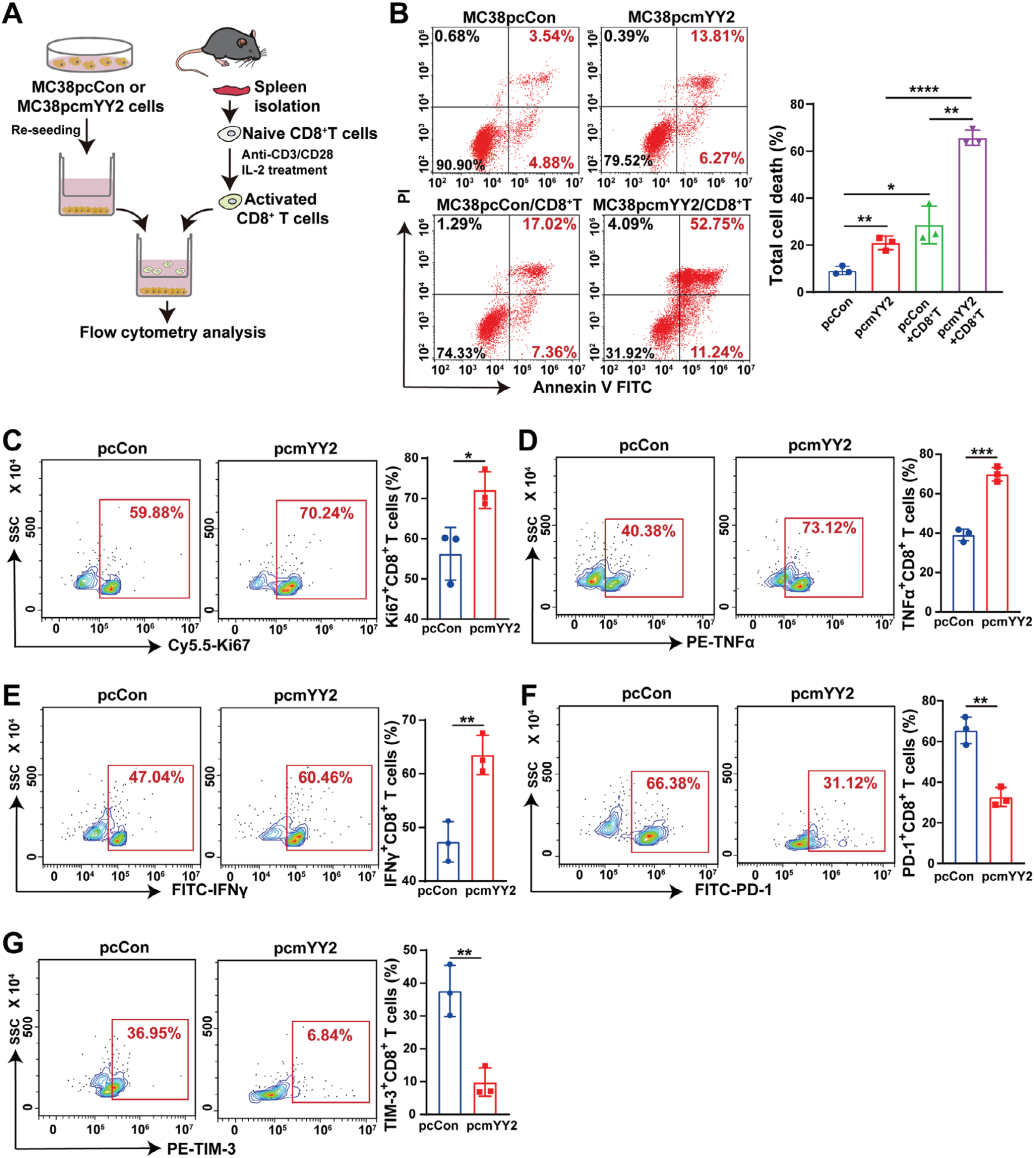
*YY2* overexpression‐induced tumor inflammatory environment enhances CTL proliferation and activation. A) Schematic diagram showing co‐culture of MC38 tumor cells with CD8^+^ T cells. B) Total cell death rate (Annexin V^+^/PI^−^ and Annexin V^+^/PI^+^ cells) of *YY2*‐overexpressing MC38 cells co‐cultured with CD8^+^ T cells for 48 h (ratio of MC38 cells/CD8^+^ T cells: 1/5). C–G) Percentages of Ki67^+^CD8^+^ (C), TNFα^+^CD8^+^ (D), IFNγ^+^CD8^+^ (E), PD‐1^+^CD8^+^ (F), and TIM‐3^+^CD8^+^ (G) T cells after co‐culturing with *YY2*‐overexpressing MC38 cells for 48 h. Cells transfected with pcCon were used as control. Quantification data are shown as mean ± S.D. All data were obtained from three independent experiments. pcCon: pcEF9‐Puro; *p < 0.05; **p < 0.01; ***p < 0.001; ****p < 0.0001.

Next, we confirmed the effect of *YY2*‐overexpressing tumor cells on CD8^+^ T cells in vitro by co‐culturing CTLs derived from mouse spleens with different MC38 cells (**Figure** **3**A). While *YY2* overexpression alone or co‐culturing of control MC38 cells with CTLs increased the total cell death rate to ≈20%, co‐culturing MC38 cells overexpressing *YY2* with CTLs increased it to more than 60% (Figure 3B), indicating that the inflammatory environment induced by *YY2* overexpression boosted the cytotoxicity and tumor elimination capability of CTLs. Co‐culturing with MC38 cells overexpressing *YY2* also stimulated the proliferation and activation of CTLs, as indicated by abundant Ki67^+^CD8^+^, TNFα^+^CD8^+^, and IFNγ^+^CD8^+^ T cells (Figure 3C–E); while suppressing CTL exhaustion, as revealed by a decrease in PD‐1^+^CD8^+^ and TIM‐3^+^CD8^+^ T cells (Figure 3F,G). These data were in accordance with the above‐mentioned in vivo experimental results. Together, these findings demonstrated that YY2 could boost the host immune response by enhancing CTL tumor‐killing activities.

### 2.3 YY2 Overexpression Promotes Pyroptosis and CTL Activation by Increasing Micronuclei‐Induced Cytosolic dsDNA Response

**Figure 4:**
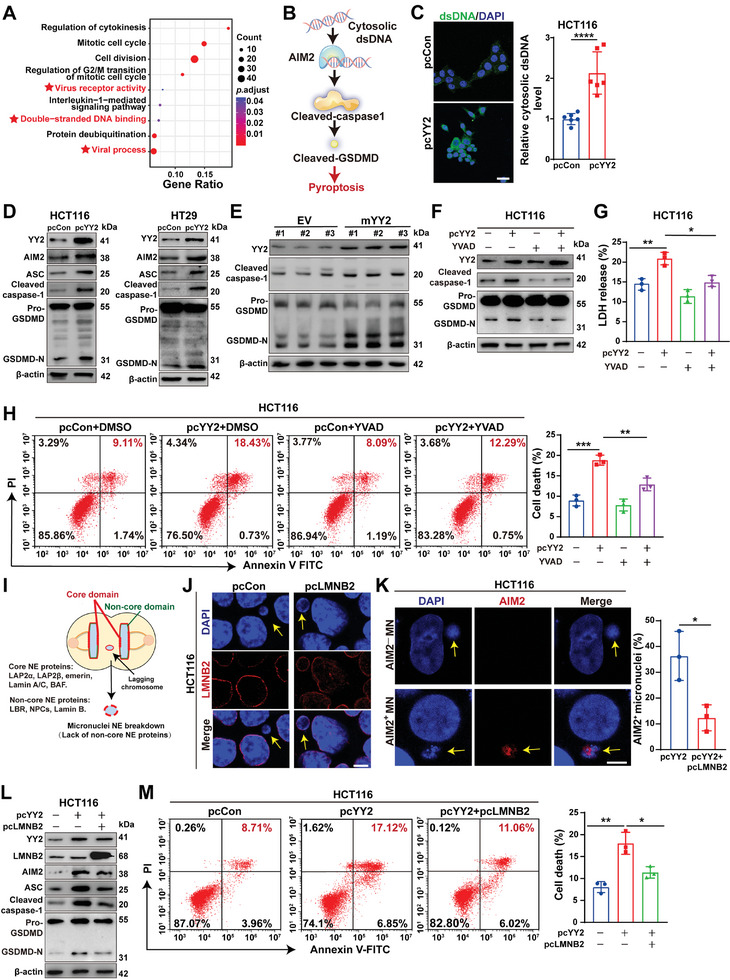
YY2‐induced micronuclei trigger pyroptosis through AIM2‐mediated cytosolic dsDNA response. A) GO enrichment analysis of DEGs in *YY2*‐overexpressing HCT116 cells obtained from RNA‐seq. B) Schematic diagram showing the molecular mechanism of AIM2‐mediated pyroptosis. C) Cytosolic dsDNA in *YY2‐*overexpressing HCT116 cells (scale bars: 100 µm). D,E) Expression levels of AIM2 pathway‐related proteins in *YY2*‐overexpressing CRC cells (D) and syngeneic graft tumor lesions formed by *YY2*‐overexpressing MC38 cells (E). F) Expression levels of cleaved caspase‐1 and GSDMD‐N in *YY2*‐overexpressing HCT116 cells treated with Z‐YVAD‐FMK for 48 h (final concentration: 10 µM). G,H) LDH release from (G) and cell death rate in (H) *YY2*‐overexpressing HCT116 cells treated with Z‐YVAD‐FMK for 48 h (final concentration: 10 µm). I) Schematic diagram showing micronucleus structure. J) Localization of LMNB2 detected by immunofluorescence staining (indicated by arrow, scale bar: 50 µm). K) AIM2‐positive micronucleus rate in HCT116 cells, as indicated by arrows. Representative images (left panels; scale bar: 20 µm) and quantification results (right panels; each dot represents AIM2^+^ micronucleus rate/slide, total cell counted >100 micronucleated cells/slide obtained from three independent experiments) are shown. L) Expression levels of AIM2 pathway‐related proteins in *LMNB2*‐overexpressing, *YY2*‐overexpressing HCT116 cells. M) Cell death rate in *LMNB2*‐overexpressing, *YY2*‐overexpressing HCT116 cells. Cells transfected with pcCon were used as control. β‐actin was used as western blotting loading control. Quantification data are shown as mean ± S.D. All data were obtained from three independent experiments, unless otherwise indicated. pcCon: pcEF9‐Puro; NE: nuclear envelope; EV: empty lentivirus; YVAD: Z‐YVAD‐FMK; *p < 0.05; **p < 0.01; ***p < 0.001; ****p < 0.0001.

We next investigated the mechanism underlying YY2‐mediated pyroptosis. GO analysis revealed enrichments in “virus receptor activity,” “double‐stranded DNA binding,” and “viral process” in *YY2*‐overexpressing HCT116 cells (**Figure** **4**A). Cytosolic dsDNA leaked from damaged mitochondria or dead cells could be recognized as viral‐like DNA and detected by AIM2, a typical dsDNA sensor.^[^ ^31^ ^]^ AIM2 in turn interacts with apoptosis‐associated speck‐like protein containing a CARD (ASC) and activates the host defense response by cleaving pro‐GSDMD into active GSDMD‐N, which triggers pyroptosis (Figure 4B).^[^ ^32^, ^33^ ^]^ Thus, we investigated whether *YY2* induced pyroptosis by activating the cytosolic dsDNA response. *YY2* overexpression robustly increased the levels of cytosolic DNA (Figure 4C; Figure S4A, Supporting Information), AIM2, ASC, cleaved caspase‐1, and GSDMD‐N, both in cells and in syngeneic graft tumor lesions (Figure 4D,E). To confirm that *YY2‐*induced pyroptosis was caspase‐1‐dependent, we treated *YY2*‐overexpressing cells with the caspase‐1 inhibitor Z‐YVAD‐FMK. Z‐YVAD‐FMK abolished *YY2*‐induced effects (Figure 4F; Figure S4B, Supporting Information), such as LDH release and cell death rate (Figure 4G,H; Figure S4C,D, Supporting Information). Notably, *YY2* overexpression failed to significantly alter the level of caspase‐4 and caspase‐5 (Figure S4E, Supporting Information). Given that these caspases are upstream regulators of GSDMD and trigger the activation of caspase‐1‐independent GSDMD/pyroptosis, these results further confirmed that YY2‐triggered pyroptosis occurs via a caspase‐1‐dependent pathway. Furthermore, *AIM2* knockdown alleviated the expression levels of ASC as well as the activation of caspase‐1 and GSDMD, while decreasing the release of LDH and cell death rate (Figure S4F–J, Supporting Information). These results demonstrated that YY2 activates the AIM2‐mediated caspase‐1‐dependent cytosolic dsDNA response, thereby promoting an inflammatory environment and pyroptosis.

However, the origin of cytosolic dsDNA remains unclear. Given that YY2 promoted chromosome missegregation, we investigated its relationship to cytosolic dsDNA‐mediated pyroptosis. Treatment with reversine suppressed *YY2* overexpression‐induced cytosolic dsDNA levels and the AIM2/caspase‐1/GSDMD pathway (Figure S5A,B, Supporting Information). This finding hinted at the involvement of chromosome missegregation in *YY2*‐induced cytosolic dsDNA response and pyroptosis. Missegregated chromosomes will eventually lead to the formation of micronuclei, which contain extra‐chromosomal dsDNA.^[^ ^13^ ^]^ Owing to the absence of non‐core nuclear envelope proteins, micronuclei are fragile, releasing dsDNA into the cytoplasm upon their rupture (Figure 4I).^[^ ^34^ ^]^ Thus, we assessed whether cytosolic dsDNA released from micronuclei could trigger pyroptosis. Given that overexpressing *lamin B2* (*LMNB2*) could stabilize micronuclei without causing large‐scale structural alternation upon overexpression, we stabilized micronuclei membrane and prevented their rupture by overexpressing *LMNB2* as reported previously (Figure 4J).^[^ ^35^, ^36^ ^]^ *LMNB2* overexpression significantly decreased the accumulation of cytosolic dsDNA (Figure S5C, Supporting Information), AIM2^+^ micronuclei (Figure 4K), and AIM2/caspase‐1/GSDMD pathway activity in *YY2*‐overexpressing CRC cells (Figure 4L; Figure S5D, Supporting Information). Additionally, it suppressed the increase in LDH release, IL‐1β secretion (Figure S5E,F, Supporting Information), and cell death rate (Figure 4M; Figure S5G, Supporting Information). Notably, while *LMNB2* overexpression increased the number of micronuclei, it failed to significantly alter the levels of chromosome missegregation (Figure S5H,I, Supporting Information), YY2 expression, AIM2/caspase‐1/GSDMD pathway activation, and cell death (Figure S5J,K, Supporting Information). These results indicated that, while it could prevent micronuclei disruption, LMNB2 itself does not affect chromosome missegregation or pyroptosis.

Subsequently, we examined the effect of *LMNB2* overexpression on the YY2 tumor suppressive potential in vivo*. LMNB2* overexpression attenuated the YY2 tumor suppressive effect (Figure S5L, Supporting Information), while blocking caspase‐1/GSDMD activation, IL‐1β levels, and cell death rate (Figure S5M–P, Supporting Information). Therefore, YY2‐induced chromosome missegregation triggers the cytosolic dsDNA response and, consequently, suppresses the tumorigenic potential by inducing tumor cell pyroptosis.

Next, we evaluated the role of cytosolic dsDNA in YY2‐induced immune responses in tumor lesions. *LMNB2* overexpression significantly alleviated CTL infiltration and proliferation (Figure S6A–C, Supporting Information), as well as their activation (Figure S6D,E, Supporting Information). Furthermore, it re‐enhanced T cell exhaustion, which was suppressed by *YY2* overexpression (Figure S6F,G, Supporting Information). These results demonstrated that micronuclei, which release cytosolic dsDNA and induce pyroptosis, are crucial for YY2‐mediated CTL proliferation and activation.

Interestingly, *YY2* knockout decreased mitotic time and prevented mitotic checkpoint activation (Figure S7A,B; Videos S4 and S5, Supporting Information). Subsequently, it promoted tumor cell viability and colony formation potential, as well as chromosome missegregation and micronuclei formation (Figure S7C–F, Supporting Information). These results were in accordance with a previous study, which showed that while shortening mitotic time and promoting tumor cell proliferation, weakening mitotic checkpoint activity leads to a certain increase of CIN.^[^ ^37^ ^]^ We next gradually increased YY2 expression level in *YY2*‐knocked out cells, and found that partial restoration of YY2 expression level suppressed chromosome missegregation and micronucleus rates in these cells, while restoration of YY2 expression to basal level as in wild‐type cells leads to further decrease of chromosome missegregation and micronucleus rates to the level similar to those in wild‐type cells (Figure S7G–I, Supporting Information). Meanwhile, a significant re‐increase in chromosome missegregation and micronucleus rates were observed when *YY2* was overexpressed in HCT116^YY2KO^ cells to an excessive level compared to that in wild‐type cells (Figure S7G–I, Supporting Information). These results, along with evidence that YY2 positively regulates mitotic checkpoint activity, suggest that both the decrease and increase in YY2 expression level could induce chromosome missegregation and consequently, micronucleus rate. However, intriguingly, we did not observe a significant increase in cytosolic dsDNA, LMNB2, activation of the AIM2/caspase‐1/GSDMD pathway, LDH release, and cell death rate in HCT116^YY2KO^ cells (Figure S7J–M, Supporting Information), suggesting an increased capability of these cells to cope with cytosolic dsDNA. Indeed, we observed a significant increase in the expression of DNase family members, especially DNase2, which was suppressed upon *YY2* overexpression (Figure S7N,O, Supporting Information). *DNase2* knockdown significantly increased cytosolic dsDNA levels and cell death rate in HCT116^YY2KO^ cells, while *DNase2* overexpression in *YY2*‐overexpressing cells suppressed them (Figure S7P–S, Supporting Information). Thus, while *YY2* knockout induces chromosome missegregation, it fails to trigger pyroptosis, most likely due to stronger DNase expression, which degrades cytosolic dsDNA. It is also noteworthy that further inhibition of mitotic checkpoint activity by reversine in HCT116^YY2KO^ cells resulted in increased chromosome missegregation and micronucleus rates, ultimately leading to cell death (Figure S7T–V, Supporting Information). Together with our results showing that reversine treatment decreased mitotic checkpoint hyperactivation as well as chromosome missegregation, micronucleus rate, and cell death in *YY2*‐overexpressing cells, these results indicate that excessive chromosome missegregation level triggered by both mitotic checkpoint hypo‐ and hyperactivation would lead to tumor cell death, which are in accordance with previous report.^[^ ^13^, ^38^, ^39^ ^]^ Overall, these results suggest that whether or not chromosome missegregation can induce cell death depends not only on its level, but also on the cellular ability in coping with the stress it caused, such as the ability in degrading cytosolic dsDNA.

### 2.4 YY2 Induces Chromosome Missegregation by Promoting *BUB1B* Transcription

**Figure 5:**
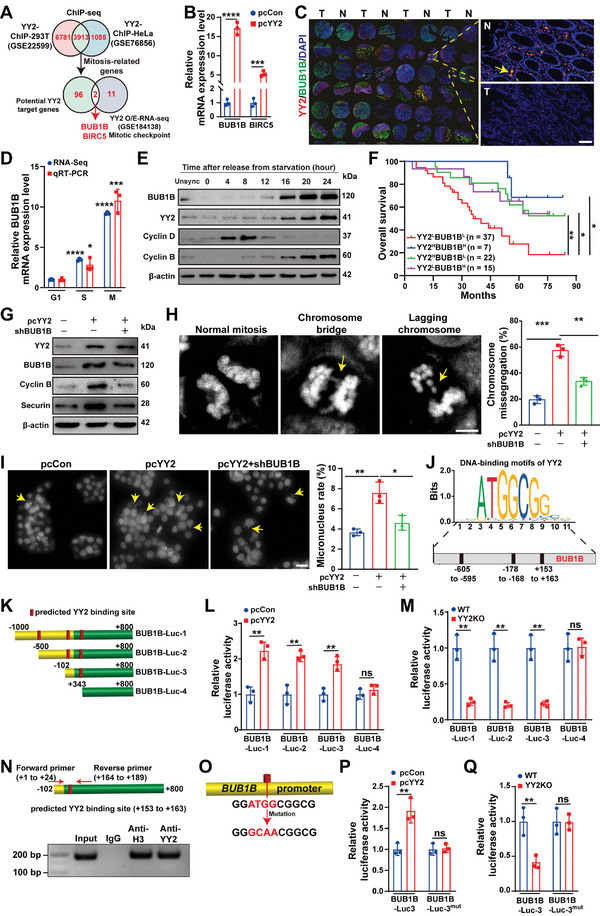
YY2 regulates chromosome segregation by enhancing *BUB1B* transcription. A) Identification of potential mitosis‐related YY2 target genes. B) *BUB1B* and *BIRC5* mRNA expression levels in *YY2‐*overexpressing HCT116 cells. C) YY2 and BUB1B expression levels in clinical CRC tissue microarrays (scale bar: 50 µm). D) *BUB1B* expression level in each cell cycle phase, as analyzed using RNA‐seq results and qRT‐PCR. Data was shown as relative to its expression in G_1_ phase. E) BUB1B and YY2 protein expression levels in HCT116 cells at indicated time points after serum starvation release. F) Kaplan‐Meier overall survival plot of CRC patients with high YY2 and high BUB1B (YY2^H^BUB1B^H^; *n* = 7), high YY2 and low BUB1B (YY2^H^BUB1B^L^; *n* = 22), low YY2 and high BUB1B (YY2^L^BUB1B^H^; *n* = 15), or low YY2 and low BUB1B (YY2^L^BUB1B^L^; *n* = 37). G) Cyclin B and securin protein levels in *BUB1B*‐knocked down, *YY2*‐overexpressing HCT116 cells. H) Percentage of cells with chromosome missegregation in *BUB1B‐*knocked down, *YY2*‐overexpressing HCT116 cells. Representative images (scale bars: 5 µm) and quantification results are shown. I) Micronucleus rate in *BUB1B‐*knocked down, *YY2*‐overexpressing HCT116 cells. Representative images of micronuclei (indicated by arrows; scale bars: 100 µm) and quantification results are shown (each dot represents micronucleus rate/slide, total cell counted >300 cells/slide obtained from 3 independent experiments). J,K) Schematic diagram of predicted YY2 binding sites on *BUB1B* promoter (J) and luciferase reporter vectors bringing the fragments of *BUB1B* promoter (K). L,M) Relative luciferase activities of *BUB1B* reporter vectors in *YY2*‐overexpressing (L) and *YY2*‐knocked out (M) HCT116 cells. N) Binding capacity of *YY2* to the predicted region in the *BUB1B* promoter. Locations of the primer pair used for PCR are shown. O–Q) Schematic diagram (O) as well as relative luciferase activities of BUB1B‐Luc‐3^mut^ in *YY2‐*overexpressing (P) and *YY2*‐knocked out HCT116 cells (Q). Cells transfected with pcCon or wild‐type HCT116 cells were used as controls. β‐actin was used for qRT‐PCR normalization and as western blotting loading control. Quantification data are shown as mean ± S.D. All data were obtained from three independent experiments, unless otherwise indicated. pcCon: pcEF9‐Puro; Unsync: unsynchronized; ns: not significant; *p < 0.05; **p < 0.01; ***p < 0.001; ****p < 0.0001.

To reveal the molecular mechanism underlying YY2‐mediated chromosome missegregation, we compared the DEGs related to mitotic checkpoint function identified in our previous study (GSE184138) from RNA‐seq of *YY2*‐overexpressing cells, with mitosis‐related genes from two chromatin immunoprecipitation (ChIP)‐seq datasets obtained from the GEO database (GSE76856 and GSE22599).^[^ ^23^, ^40^ ^]^ *BUB1B* and *baculoviral iap repeat‐containing protein 5* (*BIRC5*) were identified as potential YY2 targets (**Figure** **5**A). Notably, *BUB1B* expression increased more in *YY2*‐overexpressing HCT116 cells (Figure 5B). The increase of BUB1B expression in *YY2*‐overexpressing cells was also confirmed at protein level (Figure S8A, Supporting Information). Tissue microarrays showed that, akin to YY2, BUB1B levels were downregulated in clinical CRC tissues compared to corresponding normal adjacent tissues (Figure 5C; Figure S8B, Supporting Information). This correlation was further confirmed using a dataset obtained from GEPIA 2 (http://gepia2.cancer‐pku.cn/#index; Figure S8C, Supporting Information). Furthermore, BUB1B expression also peaked in the M phase (Figure 5D; Figure S8D,E, Supporting Information). Both YY2 and BUB1B protein levels started rising 16 h post‐starvation, when cells began their entry into G_2_/M phase, as marked by a decrease in cyclin D, a G_1_ phase cyclin, along with an increase in cyclin B, an M phase cyclin (Figure 5E; Figure S8F, Supporting Information). Moreover, as with YY2, BUB1B levels correlated positively with overall survival (Figure S8G, Supporting Information). Meanwhile, patients with low YY2 and BUB1B (YY2^L^BUB1B^L^) had the lowest survival rate; whereas those with high YY2 and BUB1B (YY2^H^BUB1B^H^) had an improved prognosis (Figure 5F).

Similar with *YY2* overexpression, *BUB1B* overexpression boosted cyclin B and securin levels, thereby prolonging mitotic time (Figure S9A,B; Videos S7 and S8, Supporting Information); while *BUB1B* knockdown had the opposite effect (Figure S9C,D; Videos S9 and S10, Supporting Information). *BUB1B* overexpression promoted chromosome missegregation and increased the percentage of micronucleated cells (Figure S9E,F, Supporting Information). Instead, *BUB1B* knockdown abolished the effect of *YY2* overexpression on mitotic checkpoint activity (Figure 5G), mitotic time (Figure S9G; Videos S11–S13, Supporting Information), number of aneuploid cells (Figure S9H, Supporting Information), chromosome missegregation (Figure 5H), and micronucleated cells (Figure 5I). Notably, compared to BUB1B inhibition, treatment with BIRC5 inhibitor sepantronium bromide only slightly reversed the effect of YY2 on chromosome missegregation and micronucleus rate, suggesting that the effect of BIRC5 on YY2‐induced chromosome missegregation was less pronounced than that of BUB1B (Figure S9I,J, Supporting Information). Together, these results revealed that BUB1B is crucial for YY2‐induced mitotic checkpoint hyperactivation and chromosome missegregation.

To examine whether YY2 promoted directly *BUB1B* transcriptional activity, we predicted four potential YY2 binding sites in the ‐605 to ‐595, ‐178 to ‐168, and +153 to +163 sites of the *BUB1B* promoter using JASPAR (https://jaspar.genereg.net; Figure 5J). Accordingly, we constructed reporter vectors carrying the ‐1000 to +800 (BUB1B‐Luc‐1), ‐500 to +800 (BUB1B‐Luc‐2), ‐102 to +800 (BUB1B‐Luc‐3), and +343 to +800 (BUB1B‐Luc‐4) sites of the *BUB1B* promoter (Figure 5K). *YY2* exerted a positive effect on the transcription of BUB1B‐Luc‐1, BUB1B‐Luc‐2, and BUB1B‐Luc‐3 but not on that of BUB1B‐Luc‐4, indicating that the ‐102 to +342 site in the *BUB1B* promoter was essential for YY2 regulation of *BUB1B* transcriptional activity (Figure 5L,M). The ChIP assay revealed that YY2 bounds to the +1 to +189 site in the *BUB1B* promoter (Figure 5N), whereas mutations from ATGG to GCAA in that predicted binding site abolished the effect of *YY2* on the *BUB1B* promoter transcriptional activity (Figure 5O–Q). These results revealed that YY2 triggers mitotic checkpoint hyperactivation‐induced chromosome missegregation by directly regulating *BUB1B* transcriptional activity.

### 2.5 BUB1B is Crucial for YY2‐Induced Pyroptosis and Tumor Immune Response

**Figure 6:**
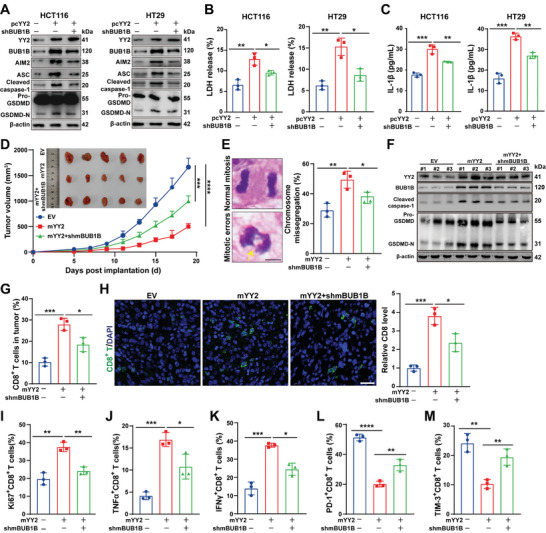
YY2/BUB1B axis triggers pyroptosis‐mediated CTL activation by enhancing micronuclei formation. A) Expression levels of AIM2 pathway‐related proteins in *BUB1B*‐knocked down, *YY2*‐overexpressing CRC cells. B,C) LDH (B) and IL‐1β (C) releases from *BUB1B*‐knocked down, *YY2*‐overexpressing CRC cells. D) Tumor volume and morphological images of syngeneic graft tumors formed by *BUB1B*‐knocked down, *YY2*‐overexpressing MC38 cells (*n* = 5/group). E) Percentage of cells with chromosome missegregation in the syngeneic graft tumor lesions formed by *BUB1B*‐knocked down, *YY2*‐overexpressing MC38 cells. Representative images (scale bars: 5 µm) and quantification results are shown (each dot represents one slide, total cell counted >100 mitotic cells/group). F) Expression levels of AIM2 pathway‐related proteins in the syngeneic graft tumor lesions formed by *BUB1B*‐knocked down, *YY2*‐overexpressing MC38 cells. G,H) Percentages of CD8^+^ T cells in the syngeneic graft tumor lesions formed by *BUB1B*‐knocked down, *YY2*‐overexpressing MC38 cells, as examined using flow cytometry (G; three tumors from three mice for each group) and immunofluorescent staining (H; left panels: representative images, scale bars: 200 µm; right panels: quantification results, each dot represents quantification results of two slides from the same mice, total 6 slides from 3 mice). I–M) Percentages of Ki67^+^CD8^+^ (I), TNFα^+^CD8^+^ (J), IFNγ^+^CD8^+^ (K), PD‐1^+^CD8^+^ (L), and TIM‐3^+^CD8^+^ (M) T cells in the syngeneic graft tumor lesions formed by *BUB1B*‐knocked down, *YY2*‐overexpressing MC38 cells (three tumors from three mice for each group). Cells transfected with pcCon were used as control. β‐actin was used as western blotting loading control. Quantification data are shown as mean ± S.D. All data were obtained from three independent experiments, unless otherwise indicated. pcCon: pcEF9‐Puro; EV: empty lentivirus; *p < 0.05; **p < 0.01; ***p < 0.001; ****p < 0.0001.

Next, we explored whether BUB1B‐mediated chromosome missegregation was involved in YY2‐induced cytosolic dsDNA response, pyroptosis, and tumor immune response. *BUB1B* knockdown in HCT116 and HT29 cells canceled the induction of cytosolic dsDNA (Figure S10A, Supporting Information) and the AIM2/caspase‐1/GSDMD pathway (**Figure** **6**A). Furthermore, it prevented the decrease in cell viability and rise in cell death (Figure S10B–E, Supporting Information), as well as increase in LDH and IL‐1β release (Figure 6B,C). Syngeneic graft experiments with MC38 cells overexpressing *YY2* showed that *BUB1B* knockdown abolished the YY2 tumor suppressive effect (Figure 6D), as well as its positive impact on chromosome missegregation, caspase‐1/GSDMD activation, IL‐1β, and cell death rate in syngeneic graft lesions (Figure 6E,F; Figure S10F–H, Supporting Information). These results revealed that BUB1B is crucial for YY2‐mediated chromosome missegregation, as well as consequent cytosolic dsDNA response and pyroptosis.

Moreover, we explored whether BUB1B was involved in YY2‐induced anti‐tumor immune response. *BUB1B* knockdown in *YY2*‐overexpressing MC38 cells suppressed the increase in CTL infiltration (Figure 6G,H), proliferation, and activation, while restoring T cell exhaustion. In particular, it suppressed Ki67^+^CD8^+^, TNFα^+^CD8^+^, and IFNγ^+^CD8^+^ T cells while restoring PD‐1^+^CD8^+^ and TIM‐3^+^CD8^+^ T cells in tumor lesions (Figure 6I–M). These results indicated that the YY2/BUB1B axis is crucial for promoting CTL proliferation and activation in tumor lesions.

Together, these results highlight the pivotal role of BUB1B in YY2‐induced cellular and immune responses. BUB1B is essential for YY2‐mediated chromosome missegregation, which triggers cytosolic dsDNA response and pyroptosis. Moreover, the YY2/BUB1B axis is crucial for promoting CTL proliferation and activation within tumor lesions, thereby enhancing the anti‐tumor immune response. This dual functionality of the YY2/BUB1B axis in both pyroptosis and immune activation underscores its potential as a promising immunotherapy target.

### 2.6 YY2 Overexpression Enhances the Efficacy of Immunotherapy

**Figure 7:**
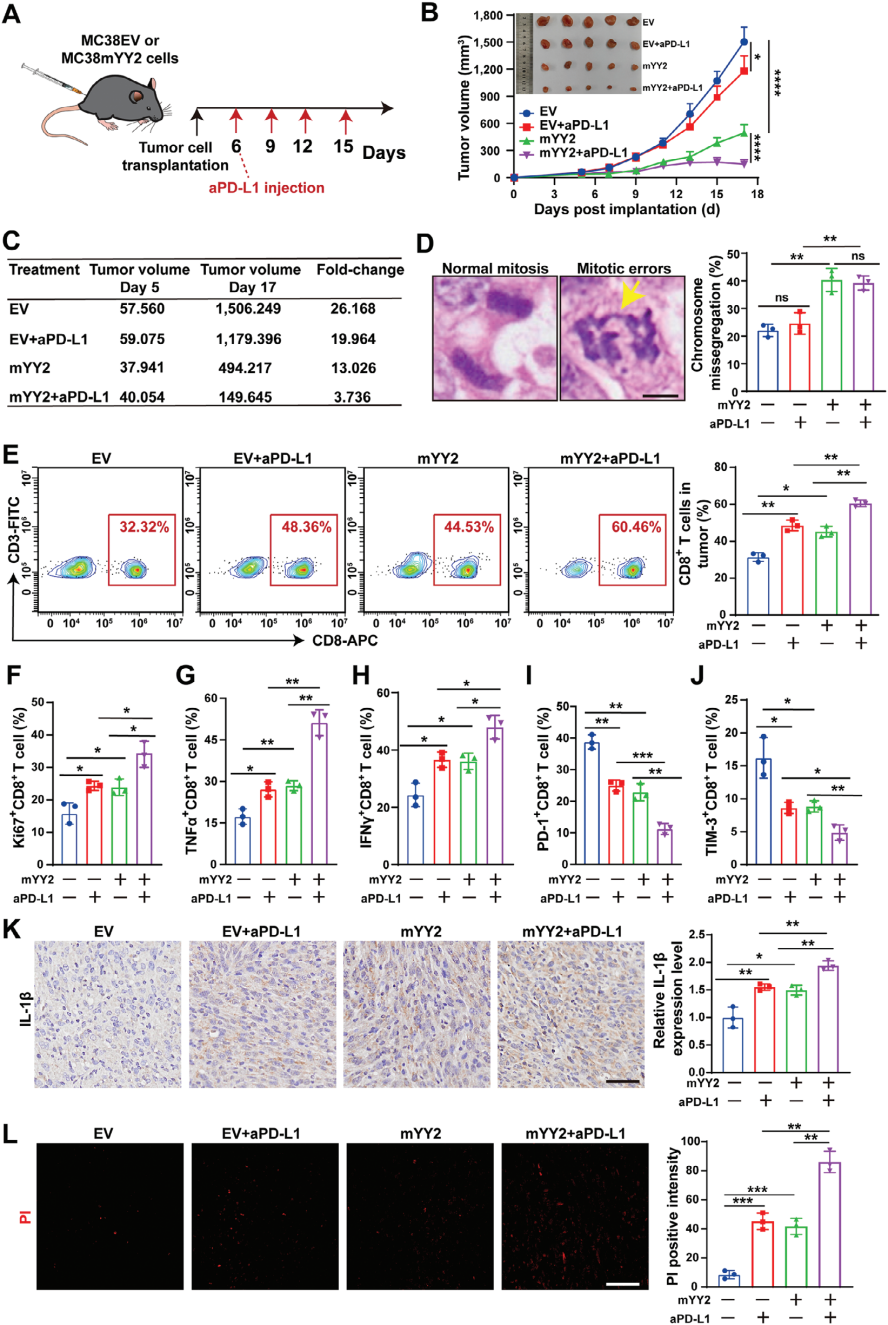
*YY2* overexpression enhances MSI CRC sensitivity to anti‐PD‐L1 antibody. A) Schematic diagram showing the combinatorial therapy procedure in MSI CRC mouse model. B) Tumor volume and morphological images of the syngeneic graft tumors formed by *YY2*‐overexpressing MC38 cells and treated with anti‐PD‐L1 antibody at indicated time‐points (final concentration: 10 mg kg^−1^ bodyweight; *n* = 5/group). C) Fold‐change of syngeneic graft tumor volumes at day 17 compared to those at the starting point of treatment (day 5). D) Percentage of cells with chromosome missegregation in the syngeneic graft tumor lesions formed by *YY2*‐overexpressing MC38 cells and treated with anti‐PD‐L1 antibody. Representative images of chromosome bridge (indicated by arrows; scale bars: 5 µm) and quantification results (each dot represents one slide, total cell counted >100 mitotic‐cells/group) are shown. E) Percentages of CD8^+^ T cells in the syngeneic graft tumor lesions (three tumors from three mice for each group). F–J) Percentages of Ki67^+^CD8^+^ (F), TNFα^+^CD8^+^ (G), IFNγ^+^CD8^+^ (H), PD‐1^+^CD8^+^ (I), and TIM‐3^+^CD8^+^ (J) T cells in the syngeneic graft tumor lesions formed by *YY2*‐overexpressing MC38 cells and treated with anti‐PD‐L1 antibody (three tumors from three mice for each group). K,L) IL‐1β expression level (K) and cell death rate (L) in the syngeneic graft tumor lesions formed by *LMNB2*‐overexpressing, *YY2*‐overexpressing MC38 cells and treated with anti‐PD‐L1 antibody, as determined by immunohistochemical staining and in vivo PI staining. Representative images (left panels; scale bars: 200 µm) and quantification results (right panels; each dot represents quantification results of two slides from the same mice, total 6 slides from 3 mice) are shown. Quantification data are shown as mean ± S.D. EV: empty lentivirus; ns: not significant; *p < 0.05; **p < 0.01; ***p < 0.001; ****p < 0.0001.

ICI therapy blocks the engagement of PD‐L1 with its co‐inhibitory receptor PD‐1, reactivating and expanding tumor‐reactive CTLs.^[^ ^41^ ^]^ Instead, pyroptosis‐enhanced CTL activation exerted a synergistic effect by promoting tumor cell sensitivity to ICI therapy.^[^ ^42^ ^]^ However, the efficacy of ICI therapy in solid tumors such as CRC is lower than expected, and is even lower in MSS tumors.^[^ ^43^ ^]^ Therefore, we explored whether YY2‐mediated CTL activation favored MSI and MSS CRCs sensitivity to ICI immunotherapy (**Figure** **7**A; Figure S11A, Supporting Information). Treatment with the anti‐PD‐L1 antibody slightly suppressed the growth of syngeneic graft tumors formed by MSI MC38 cells (Figure 7B); however, it did not exert any significant effect on those formed by MSS CT26 cells (Figure S11B, Supporting Information). Meanwhile, combination of anti‐PD‐L1 antibody with *YY2* overexpression significantly reduced the tumor volume (Figure 7B; Figure S11B, Supporting Information). Compared to the corresponding controls, which increased more than 26‐fold and 32‐fold within 12 days, *YY2* overexpression treatment alone limited the growth of both MSI and MSS CRC tumors to 13‐fold or 18‐fold, respectively. Treatment by merely anti‐PD‐L1 antibody, while could limit the MSI CRC tumor growth to 19‐fold, failed to suppress the growth of MSS CRC tumors, confirming that MSS CRC tumors were not sensitive to ICI therapy. However, combination of *YY2* overexpression and anti‐PDL‐L1 therapy reduced both MSI and MSS CRC tumor growth to less than four and sixfold, respectively (Figure 7C; Figure S11C, Supporting Information). Moreover, *YY2* overexpression, which resulted in a higher rate of chromosome missegregation (Figure 7D; Figure S11D, Supporting Information), further increased anti‐PD‐L1 antibody treatment‐induced CTL infiltration into tumor lesions formed by MSI or MSS CRC cells (Figure 7E; Figure S11E,F, Supporting Information). Similarly, this combination increased the ratios of Ki67^+^CD8^+^, TNFα^+^CD8^+^, and IFNγ^+^CD8^+^ T cells (Figure 7F–H; Figure S11G–I, Supporting Information); while reducing those of PD‐1^+^CD8^+^ and TIM‐3^+^CD8^+^ T cells (Figure 7I,J; Figure S11J,K, Supporting Information). We observed a significant increase in IL‐1β expression and cell death in both MSI and MSS tumor lesions overexpressing *YY2* and treated with anti‐PD‐L1 antibody (Figure 7K,L; Figure S11L, M, Supporting Information). These results revealed that *YY2* overexpression/anti‐PD‐L1 combinatorial therapy induces chromosome missegregation, pyroptosis, and the development of the immune microenvironment in tumor lesions, triggering CTL proliferation and activation while preventing their exhaustion. Consequently, CTLs enhance their tumor‐killing effect in both MSI and MSS tumors.

Taken together, our results demonstrated that YY2 promotes the formation of micronuclei and the release of dsDNA by enhancing BUB1B expression and chromosome missegregation, thereby inducing pyroptosis and the development of a tumor immune microenvironment. This boosts CTL activity and, consequently, the effect of immunotherapy on CRC cells.

## 3 Discussion

Immune system disorders and chromosome missegregation are major hallmarks of cancer, which play important roles in tumor development.^[^ ^41^, ^44^ ^]^ Our results unravel a novel, unprecedented link between these events. Specifically, we reveal that pyroptosis, triggered by chromosome missegregation‐induced cytosolic dsDNA, increases tumor inflammation, thereby enhancing CTL infiltration and cytotoxicity by promoting their proliferation and activation. ICI therapy has emerged as a promising anti‐tumor immunotherapy; however, its application in solid tumors is limited due to low immune cell infiltration and suboptimal T cell activation.^[^ ^45^, ^46^ ^]^ Over 90% of patients with CRC and melanoma exhibit a hypoimmune response and a limited response to ICI treatment. This tendency is even higher in patients with MSS tumors. Thus, boosting the immune response to solid tumors is crucial for improving ICI treatment efficacy.^[^ ^47^, ^48^, ^49^ ^]^ Our results reveal that *YY2* overexpression triggers pyroptosis by inducing chromosome missegregation, which consequently enhances tumor sensitivity to anti‐PD‐L1 therapy in CRC, suggesting a potential combinatorial strategy to improve ICI therapy, especially in MSS CRC. Notably, our bioinformatics analysis uncovered the prevalence of chromosome missegregation in multiple solid tumors. Hence, while whether chromosome missegregation could also contributes to the CTL infiltration and activation through neoantigen generation or activation of other immune cells remains to be investigated, our findings suggest the possibility of extending this combinatorial strategy to solid tumors other than CRC.

According to their DNA mismatch repair protein deletion and mutation, tumors, including CRC, could be classified into MSI and MSS types. MSS tumors show effective DNA mismatch repair and are more resistant to ICI therapy.^[^ ^50^ ^]^ Meanwhile, MSI tumors, which often experience DNA mutations due to their defects in DNA mismatch repair proteins, are more sensitive to ICI therapy.^[^ ^51^ ^]^ Our findings showed that while ICI therapy using anti‐PD‐L1 antibody failed to exert any significant effect on MSS tumors, pyroptosis triggered by YY2‐induced chromosome missegregation could increase inflammatory environment in MSS tumors, thereby activating the CTL cells, and subsequently decreasing their resistance to immunotherapy. Thus, our findings propose a potential anti‐tumor immunotherapy for both MSS and MSI tumors by combining chromosome missegregation inducer and ICI.

Pyroptosis was initially discovered as immunogenic cell death in response to bacterial or viral infections.^[^ ^52^, ^53^ ^]^ However, later studies revealed that dsDNA, such as DNA leaking from damaged mitochondria and dead cells, could also trigger pyroptosis.^[^ ^54^, ^55^ ^]^ Micronuclei, a product of chromosome missegregation, are fragile due to the absence of non‐core nuclear envelope proteins and, thereby, can release their dsDNA into the cytosol. However, whether cytosolic dsDNA originating from micronuclei can cause pyroptosis has not been reported. Here, we show that DNA fragments released from micronuclei triggered a cytosolic dsDNA‐mediated inflammatory response and, subsequently, pyroptosis. These findings not only reveal a novel regulatory mechanism of tumor cell pyroptosis by YY2, but also demonstrate, for the first time, that cytosolic dsDNA released from micronuclei could be detected by AIM2, thereby activating the AIM2/caspase‐1/GSDMD pathway. This, in turn, triggers pyroptosis, enhancing the tumor inflammation environment, followed by increased CTL proliferation and activation. Thus, our findings provide novel insights into the consequences of micronuclei formation in tumor cells, as well as the regulation of AIM2‐mediated cytosolic dsDNA response.

The mitotic checkpoint serves as a surveillance mechanism that guarantees proper chromosome segregation and the correct inheritance of genetic information.^[^ ^56^ ^]^ Impaired mitotic checkpoint activity leads to chromosome missegregation, which could subsequently trigger CIN. However, our understanding regarding the mechanisms of mitotic checkpoint regulation, as well as the consequent effect of chromosome missegregation in tumor cells remains limited. Through cross‐omics analysis, we identified YY2 as a novel M‐phase regulator, whose expression oscillates with cell cycle progression. Furthermore, we show that YY2/BUB1B axis‐induced mitotic checkpoint hyperactivation triggers chromosome missegregation and cytosolic dsDNA, thereby inducing cytosolic dsDNA response and promoting tumor immunity. YY2 downregulation has been found in various cancers and is correlated with poor prognosis, thus, our findings demonstrate novel mechanism underlying its tumor suppressive effect. However, how YY2 is regulated during cell cycle and in tumor cells, as well as whether its epigenetic and/or post‐translational regulation are involved, needs further investigations.

Although harmful to non‐cancerous cells, a certain level of CIN is tolerated by tumor cells. Indeed, a certain increase in CIN can enhance tumor heterogeneity and fitness, and correlates with poor prognosis.^[^ ^7^ ^]^ Our current findings show that YY2 could promote transcription of *BUB1B*, which is involved in assuring proper mitotic checkpoint activity and chromosome segregation. Interestingly, akin to overexpression, *YY2* downregulation could also enhance micronuclei formation without significant impact on cell death. While further studies are required to ascertain the involvement of other pathways, our findings show that higher DNase levels are crucial for increasing tolerance to chromosome missegregation and the threshold for cytosolic dsDNA‐induced tumor cell death. Our results show that YY2 is a mitotic checkpoint regulator and, therefore, adjusting chromosome missegregation by controlling YY2 expression is a potential anti‐tumor strategy.

In conclusion, our study unraveled a novel pyroptosis regulatory mechanism triggered by an AIM2‐mediated response activated by cytosolic dsDNA originating from micronuclei. This, in turn, promotes T cell proliferation and activation, boosting the efficacy of ICI therapy. We also identified the YY2/BUB1B axis as a novel regulator of chromosome missegregation that leads to the formation of micronuclei. Thus, our study not only provides new insights into the molecular mechanism of pyroptosis and the consequences of chromosome missegregation in tumors, but unveils also a novel function of YY2. Finally, while further preclinical and clinical studies are necessary, for example for controlling the levels of chromosome missegregation to avoid the production of carcinogenic CIN are necessary, our findings suggest a potential anti‐tumor therapy for both MSI and MSS cancers by combining chromosome missegregation‐inducing agents with ICI.

## 4 Experimental Section

### Vectors and Constructs

shRNA expression vectors targeting two different sites of *BUB1B, DNase2*, and *AIM2* were constructed with target sequences as follows: 5′‐GGA GGG AGA TGA ATG GGA A‐3′ (human shBUB1B‐1), 5′‐GAA GGA AAT TGA ATT AGG T‐3′ (human shBUB1B‐2), 5′‐GCA GAA ACG GGC ATT TGA A‐3′ (mouse shBUB1B‐1), 5′‐GCT GAA AGA ACG AAG GGA A‐3′ (mouse shBUB1B‐2), 5′‐GCA CAG AGG ACC ACT CCA A‐3′ (human shDNase2‐1), 5′‐GCC AGA GGA TCG ATT GAA C‐3′ (human shDNase2‐2), 5′‐CCC GCT GAA CAT TAT CAG AAA‐3′ (human shAIM2‐1), and 5′‐CTG GAG TTC ATA GCA CCA TAA‐3′ (human shAIM2‐2). Oligonucleotides containing the target sequence and its complementary sequence, as well as loop sequence, were annealed and inserted into a shRNA overexpression vector with U6 promoter and puromycin resistant gene (pcPUR+U6icassette).^[^ ^57^ ^]^ Human *YY2* (NM_206923.4; pcYY2) overexpression vector was constructed as described previously.^[^ ^23^ ^]^ For mouse *YY2* (NM_001098723.1; pcmYY2), human *BUB1B* (NM_002322.6; pcBUB1B), human *Lamin B2* (NM_032737.4; pcLMNB2), mouse *Lamin B2* (NM_010722.5; pcmLMNB2), and human *DNAse2* (NM_001375.3; pcDNase2) overexpression vectors, coding sequences were amplified from corresponding cDNA using PrimeSTAR Max DNA Polymerase (Takara Bio, Dalian, China). The human and mouse cDNAs were obtained by reverse‐transcribing 1 µg total RNA extracted from HCT116 and MC38 cells, respectively, using the PrimeScript RT Reagent Kit with gDNA Eraser (Takara Bio). The coding regions of human *YY2*, mouse *YY2*, and human *LMNB2* were inserted into the *BamH*I and *EcoR*I sites of pcEF9‐Puro vector.^[^ ^58^ ^]^ The coding regions of human *BUB1B* and human *DNAse2* were inserted into the *BamH*I and *Not*I sites, while that of mouse *LMNB2* was inserted into the *Mlu*I and *Not*I sites of pcEF9‐Puro vector.

For reporter vectors bringing different regions of *BUB1B* promoter (BUB1B‐Luc‐1 with the ‐1000 to +800 regions, BUB1B‐Luc‐2 with the ‐500 to +800 regions, BUB1B‐Luc‐3 with the ‐102 to +800 regions, and BUB1B‐Luc‐4 with the +343 to +800 regions), the corresponding regions of the *BUB1B* promoter were cloned into *Nhe*I and *Hind*III sites of the pGL4.13 (Promega, Madison, WI). Human genome DNA extracted from HCT116 cells using the TIANamp Genomic DNA Kit (Tiangen Biotech, Beijing, China) was used as template for amplifying the promoter regions. BUB1B‐luciferase reporter vector with mutated predicted YY2 binding site (BUB1B‐Luc‐3^mut^) was constructed based on the site‐specific mutagenesis method using a Site‐directed Gene Mutagenesis Kit (Beyotime Biotechnology, Shanghai, China).

Lentivirus vector overexpressing *YY2* (pLenti‐YY2) was constructed as described previously.^[^ ^23^ ^]^ Lentivirus vector overexpressing mouse *YY2* (pLenti‐mYY2) and mouse *LMNB2* (pLenti‐mLMNB2) were constructed by inserting the corresponding coding sequences into the *Bam*HI and *Xho*I sites or *Mlu*I and *Xho*I sites of the pCDH‐CMV‐MCS‐EF1‐Puro vector, respectively.

### Cell Lines and Cell Experiments

HEK293T (catalog number: GNhu17), as well as human CRC cell lines HCT116 (catalog number: TCHu 99) and HT29 (catalog number: SCSP‐ 5032) were purchased from the Cell Bank of Chinese Academy of Sciences (Shanghai, China). Mouse CRC cell lines MC38 (catalog number: IM‐M006) and CT26 (catalog number: IM‐M007) were purchased from IMMOCELL (Xiamen, China). Cells were cultured in Dulbecco's modified Eagle's medium (Gibco, Life Technologies, Grand Island, NY) with 10% FBS (Biological Industries, Beith Haemek, Israel) and 1% penicillin‐streptomycin. Cell lines were verified using short‐tandem repeat profiling method and were tested periodically for mycoplasma contamination using Mycoplasma PCR Detection Kit (Beyotime Biotechnology).

For gene transfection experiments, cells were seeded in a 6‐well plate and transfected with 2 µg of indicated vectors. Twenty‐four hours after transfection, transfected cells were selected using 1 µg mL^−1^ puromycin for 36 h. *YY2*‐knocked out HCT116 (HCT116^YY2KO^) cells were established using CRISPR/Cas9 method as described previously.^[^ ^23^ ^]^ Deletion of nucleotides located in +95 to + 151 regions (56 bp) was confirmed by sequencing. All transfections were performed using Lipofectamine 2000 (Invitrogen Life Technologies, Carlsbad, CA) according to the manufacturer's protocol.

Lentiviruses for establishing stable cell lines overexpressing mouse *YY2* and/or mouse *LMNB2* for syngeneic graft experiments were generated by co‐transfecting HEK293T cells with 8 µg pLenti‐mYY2 or pLenti‐mLMNB2 vectors, 6 µg pCMVDR, and 2 µg pCMV‐VSVG in a 10 cm plate. Culture media was changed the following day and lentivirus‐containing supernatant was harvested and filtered using a 0.45‐µm filter after 48 h. For infection, MC38 and CT26 cells were cultured in a 6‐well plate. Twenty‐four hours later, the medium was changed to 1 mL fresh culture medium and 1 mL corresponding lentivirus supernatant. Infected cells were then selected using 1 µg mL^−1^ puromycin for 7 days.

For reversine and sepantronium bromide treatment, cells were prepared as described above and treated with reversine (MedChemExpress, Shanghai, China; final concentration: 0.2 μM) or sepantronium bromide (MedChemExpress; final concentration: 1 μM), respectively, for 24 h. For Z‐YVAD‐FMK treatment, cells were prepared as described above and treated with Z‐YVAD‐FMK (MedChemExpress; final concentration: 10 μM) for 48 h.

### Clinical Human CRC Specimens

Clinical human CRC specimens were obtained from patients undergoing surgery at Chongqing University Cancer Hospital (Chongqing, China). The specimens were snap‐frozen in liquid nitrogen and stored in Biological Specimen Bank of Chongqing University Cancer Hospital. Patients did not receive chemotherapy, radiotherapy, or other adjuvant therapies prior to the surgery. Prior patient's written informed concerns were obtained. The experiments were approved by the Institutional Research Ethics Committee of Chongqing University Cancer Hospital (Permit No. CZLS2021292‐A), and conducted in accordance with Declaration of Helsinki.

### Syngeneic Graft Experiments and Treatment

C57BL/6 mice (strain number No. 000664, male, body weight: 18–22 g, 4–5 weeks old) and Balb/c mice (strain number No. N000020, male, body weight: 18–22 g, 4–5 weeks old) were purchased from the Chongqing Medical University (Chongqing, China). Mice were randomly assigned to different groups and injected subcutaneously with 1 × 10^6^ of indicated cells. Tumor size (V) was evaluated by a caliper every 2 days using the following equation: V = a × b^2^/2, where a and b are the major and minor axes of the tumor, respectively. Investigator was blinded to the group allocation and during the assessment. Animal studies were approved by the Institutional Ethics Committee of Chongqing University Cancer Hospital (Permit No. SYXK‐2021‐0001), and carried out in the Chongqing University Cancer Hospital. All animal experiments conformed to the approved Guidelines for the Care and Use of Laboratory Animals of Chongqing University Cancer Hospital. All efforts were made to minimize suffering.

For assessing the efficacy of anti‐PD‐L1 antibody treatment on MSI tumor, MC38 cells were injected subcutaneously into C57BL/6 mice as described above. After the tumor volume reached 50 mm^3^, the mice were treated with 10 mg kg^−1^ bodyweight anti‐PD‐L1 antibody (Bio X Cell, Lebanon, NH) through intraperitoneal injection every 3 days starting on day 6 after tumor transplantation for a total of four treatments. Mice treated with anti‐IgG2 isotype (Bio X Cell) were used as controls. For assessing the efficacy of anti‐PD‐L1 antibody treatment on MSS tumor, CT26 cells were injected subcutaneously into Balb/c mice as described above. Mice were then treated with anti‐PD‐L1 antibody as described above.

### scRNA‐seq

Tumors were minced and digested with 1 mg mL^−1^ Collagenase Type IV (Solarbio, Beijing, China) supplemented with 10 U mL^−1^ DNase I (Solarbio) for 1 h at 37 °C with 60 rpm. Lysates were then strained with a 70‐µm cell strainer before red blood cells were removed by suspending the strained lysates with red blood lysis buffer to obtain single‐cell suspensions. scRNA‐seq libraries were then prepared using DNBelab C Series Single‐Cell Library Prep set (MGI, 1000021082, Shenzhen, China) as previously described.^[^ ^59^ ^]^ In brief, single‐cell suspensions were converted to barcoded scRNA‐seq libraries through droplet encapsulation, emulsion breakage, mRNA captured bead collection, reverse transcription, cDNA amplification, and purification. Indexed sequencing libraries were constructed according to the manufacturer's protocol. The sequencing library concentration was quantified using a Qubit ssDNA Assay kit (Thermo Fisher Scientific, Waltham, MA). The resulting libraries were then sequenced using a DIPSEQ T1 sequencer at China National GeneBank Database (CNGBdb). Raw sequencing reads from DIPSEQ‐T1 were filtered and demultiplexed using PISA (v.0.2) (https://github.com/shiquan/PISA). Reads were aligned to genome using STAR (v.2.7.4a) and sorted by using Sambamba (v.0.7.0). Cell versus gene unique molecule identifier (UMI) count matrices were generated by a custom script. Count matrices were processed using the Seurat R (v.3.6.3) package (v.3.1.4).^[^ ^60^ ^]^ Outlier cells were filtered out based on the distribution of the counts of RNA features and expression of mitochondrial genes. Cells with fewer than 200 genes detected, greater than 12000 genes detected, or more than 10% mitochondrial reads were removed.

### RNA‐seq

RNA extraction and RNA‐seq were conducted at Novogene Bio Technology Corporation (Beijing, China) using Illumina HiSeq 2500 (Illumina, San Diego, CA; three replicates per group). Sequencing raw reads were pre‐processed by removing rRNA reads, shorts‐fragment reads, sequencing adapters, and other low‐quality reads. The cleaned reads were mapped to the human reference genome ensemble GRCh38 (hg38) with two mismatches using Tophat v2.1.0. Following the genome mapping, Cufflinks v2.1.1 was run with a reference annotation to generate FPKM values for known gene models. DEGs were then identified using Cuffdiff. The *p*‐value significance threshold in multiple tests was set by the false discovery rate (FDR). The fold‐changes were additionally calculated according to the FPKM in each sample. To identify DEGs, pairwise comparisons between M phase and G_1_ phase, as well as M phase and S phase were performed to identify genes upregulated or downregulated with an FDR ≤ 0.001 relative to each control.

### CD8^+^ T Cell Tumor Infiltration and Functional Analysis

Tumor tissues from syngeneic graft experiments were minced and digested as described above. To measure the activity of CD8^+^ T cells, the isolated cells were fixed and permeabilized with the Fixation/Permeabilization Solution Kit (Multi Sciences, Hangzhou, China) prior to incubations with antibodies. Cells stained with isotype‐matched control were used as negative controls. Antibodies used were listed in Table S1 (Supporting Information). Data was acquired using CytoFlexLX (Beckman Coulter, Indianapolis, IN) and analyzed using CytExpert.

### Mouse CD8^+^ T Cell Purification and Cytotoxicity Assays

CD8^+^ T cells isolated from the spleens of C57BL/6 mice (6‐8 weeks old) were purified by MojoSort Mouse CD8^+^ T Cell Isolation Kit (Biolegend, San Diego, CA). Naïve CD8^+^ T cells were then activated using plate‐bound a‐CD3 (5 µg mL^−1^, Biolegend) and soluble a‐CD28 (1 µg mL^−1^, Biolegend) for 48 h. CD8^+^ T cells were then expanded in the presence of IL‐2 (20 ng mL^−1^, PeproTech, Rocky Hill, NJ) with indicated cells for another 2 days. Cells were then fixed, permeabilized, and stained with the indicated antibodies prior to flow cytometry analysis using CytoFlexLX (Beckman Coulter) as described above. Antibodies used are listed in Table S1 (Supporting Information).

### Live‐Cell Imaging

For fluorescence imaging of mitotic division, cells were prepared as described above prior to seeding in a 3.5‐cm glass bottom plate (Wuxi NEST Biotechnology, Wuxi, China; 1 × 10^5^ cells per plate) and grown overnight. Nuclei were stained with Hoechst 33342 (Solarbio) for 30 min prior to imaging. The culture was maintained at 37 °C under 5% CO_2_ in a stage‐top incubator (Tokai Hit, Shizuoka, Japan). Images were acquired every 5 min for 8 h with a 20‐ms exposure time using an inverted fluorescence microscope Olympus IX83 (Olympus, Tokyo, Japan). Images were processed using Fluoview (v.2.3). Mitotic time was quantified as the time from nuclear envelope breakdown until the onset of anaphase. For cell death observation, cells were prepared as described above. After overnight incubation, culture media were changed with those containing propidium iodide (PI; 20 µg mL^−1^). Live‐cell images were acquired as described above.

### Tissue Microarray Analysis

Tissue microarray was purchased from Outdo Biotech (Shanghai, China). Samples were dewaxed using xylene. Blocking with 5% BSA was performed prior to incubation with primary antibodies followed by incubation with corresponding secondary antibodies. The results were read using Aipathwell (Servicebio, Wuhan, China) to quantify protein expression on all samples. Patients were stratified into high and low expression groups based on the relative protein expression compared to corresponding normal tissue, as determined by immunofluorescence quantification of tissue microarray samples. A value >1 indicates high expression, while a value < 1 indicates low expression. Antibodies used were listed in Table S1 (Supporting Information).

### Cell Cycle Synchronization

For synchronization using serum starvation method, cells were first synchronized in G_0_ phase by 24 h serum starvation. Cells were collected or further cultured in medium with 10% FBS for indicated time points. For synchronization using double thymidine block method, cells were treated with thymidine (final concentration: 2 μM) for 16 h. After the thymidine‐containing medium was removed, cells were washed with PBS, and cultured using medium without thymidine for 9 h. Cells were then cultured for another 14 h with thymidine to synchronize them at G_1_/S phase, Synchronized cells were then collected or further cultured in medium with 10% FBS for indicated time points. Percentages of the cells in each cell cycle phase were analyzed by PI staining followed by flow cytometry analysis using CytoFlexLX flow cytometer (Beckman Coulter).

### Metaphase Spread

Cells were arrested at metaphase by 2 µg mL^−1^ colchicine (Aladdin, Shanghai, China) treatment for 3 h before being harvested, re‐suspended in hypotonic solution (0.075  KCl), and incubated for 15 min at 37 °C. After cell swelling, cells were fixed using 2 mL of freshly prepared methanol‐acetic acid fixative (3:1) and collected by centrifugation. The pellet was re‐suspended in 5 mL of 3:1 methanol‐acetic acid for 30 min and dropped onto pre‐cooled slides. Chromosomes were then stained with DAPI (Beyotime Biotechnology) for 15 min. Images of mitotic chromosomes were acquired using fluorescence microscope (Olympus BX53). Chromosome number per cell was then quantified.

### Chromosome Bridge, Lagging Chromosome, and Micronucleus Staining

For chromosome bridge and lagging chromosome observations, cells were seeded in 3.5‐cm glass bottom plate (3 × 10^4^ cells/ plate), incubated overnight, and synchronized at prometaphase by nocodazole treatment (final concentration: 100 ng mL^−1^, Beyotime Biotechnology) for 6 h. Cells were then fixed with 4% paraformaldehyde for 15 min at room temperature. Nuclei were stained with DAPI for 15 min. Images were taken using laser scanning confocal microscopy (Leica Microsystems TCS SP5, Heidelberg, Germany), and quantification was performed by LAS X software (Leica Microsystems). For observation of chromosome bridge and lagging chromosome in clinical samples and syngeneic graft tumor lesions, fresh tissues were fixed in 4% paraformaldehyde overnight, embedded in paraffin, and sectioned at 4 µm thickness using a cryostat. After being dewaxed using xylene and rehydrated, sections were stained with 0.5% hematoxylin‐eosin (Sangon Bio, Shanghai, China). After samples were dehydrated and mounted in coverslip, images were taken using Olympus VS120 slide scanner (Olympus).

For micronuclei observation, cells were seeded in the 24‐well plate, incubated for 24 h, and stained with DAPI as described above. Images were taken using Olympus IX71 (Olympus). Micronucleus rate was defined as the ratio of total micronuclei to the total cell number.

### Immunohistochemistry, Immunofluorescence, and Hematoxylin‐Eosin (H&E) Staining

Human CRC tissues, normal adjacent tissues, and syngeneic graft tumors were fixed in 4% paraformaldehyde overnight prior to being embedded in paraffin and sectioned at 4 µm thickness using cryostat. Samples were then dewaxed using xylene, rehydrated, and subjected to staining. For H&E staining, paraffin sections were fixed with 10% formalin and washed with 60% propylene glycerol. Samples were then stained with 0.5% H&E (Solarbio) for 3 min followed by dehydration and coverslip mounting. For IHC, the tissue sections were incubated with primary antibodies for 1 h followed by incubation with the corresponding horse‐radish peroxidase‐conjugated secondary antibodies. Visualization was performed using a DAB Kit (DAKO, Beijing, China) under microscope. Nuclei were counterstained with hematoxylin (Solarbio), and the sections were then dehydrated and mounted with coverslips. Images were taken using Olympus vs120 slide scanner (Olympus). Antibodies used were listed in Table S1 (Supporting Information).

### Cytosolic dsDNA Detection

Cells were prepared as described above, seeded into 14 mm coverslips (6 × 10^4^ cells/well), and cultured for 48 h. Cells were then fixed using 4% paraformaldehyde for 15 min, and permeabilized using saponin for 5 min at room temperature. Blocking with 5% BSA was conducted prior to incubation with dsDNA antibodies (Novus Biologicals, Centennial, CO). After incubation with corresponding secondary antibody, nuclei were stained with DAPI (Beyotime Biotechnology). Images were taken and analyzed using laser scanning confocal microscopy (Leica Microsystems TCS SP5).

### ChIP Assay

Chromatin was immunoprecipitated using ChIP Assay Kit (Beyotime Biotechnology) according to the manufacturer's protocol. Briefly, cells were lysed and chromatins were then immunoprecipitated using protein A+G Agarose/Salmon Sperm DNA and anti‐YY2 antibody or normal mouse IgG, de‐crosslinked for 4 h at 65 °C, and treated with 0.5 M EDTA, 1 M Tris (pH 6.5), and 20 mg mL^−1^ proteinase K. Immunoprecipitated chromatin was then subjected to PCR using PrimeSTAR Max (Takara Bio). Primer sequences for amplifying the *BUB1B* promoter region with the predicted YY2 binding site were: 5′‐GTG TGG GCT TGA GGT GGC CGG TTT G‐3′ (forward primer); and 5′‐ CTC AGA GCA CCC CCT TCC TTC TTC A‐3′ (reverse primer).

### Dual Luciferase Reporter Assay

Cells were prepared as described above and seeded into 24‐well plate (8 × 10^4^ cells/well). 24 h later, cells were co‐transfected with indicated vectors, reporter vectors, and *Renilla* luciferase expression vector (pRL‐SV40, Promega) as the internal control. 48 h after co‐transfection, luciferase activities were measured using Dual Luciferase Assay System (Promega). Firefly luciferase activities were normalized with the corresponding *Renilla* luciferase activities.

### Cell Viability and Colony Formation Assay

Cells were prepared as described above. For cell viability assay, cells were re‐seeded into 96‐well plate (5 × 10^3^ cells/well). Cell numbers were measured by colorimetric assays with MTS (Promega) at indicated time points. For colony formation assay, cells were seeded into the 6‐well plate at a density of 300 cells per well, culture for 12 days, fixed with 4% paraformaldehyde, and stained with crystal violet. Quantification was performed by counting the number of colonies formed.

### LDH and IL‐1β Assay

Cells were prepared as described above and cultured in 24‐well plate (6 × 10^4^ cells/well) for 48 h. The supernatants were collected, and the levels of the LDH and IL‐1β were measured using LDH Cytotoxicity Assay Kit (Beyotime Biotechnology) and IL‐1β Assay Kit (ABclonal, Wuhan, China) according to the manufacturer's protocol.

### Cell Death Assay

Cells were prepared as described above and re‐seeded in a 6‐well plate (3 × 10^5^ cells/well). Cells were stained with Annexin V/PI and subjected to flow cytometry using CytoFlexLX (Beckman Coulter).

### In Vivo Cell Death Assessment

For PI labeling of cell death in vivo, mice bearing tumors formed by MC38 cells were administered with PI (final concentration: 25 mg kg^−1^) through tail‐vein injection, and euthanatized 10 min after injection. Tumors were then collected and frozen into OCT‐containing cryomold before being sectioned at 4 µm thickness using a cryostat. After mounting, the images were taken using laser scanning confocal microscopy (Leica Microsystems TCS SP5).

### RNA Extraction and qRT‐PCR

Total RNA was extracted using Trizol (Invitrogen Life Technologies) according to the manufacturer's protocol. Total RNA (1 µg) was then reverse‐transcribed into cDNA using PrimeScript Reagent Kit with gDNA Eraser (Takara Bio). qRT‐PCR was performed using SYBR Premix ExTaq (Takara Bio). The sequences of the primers used were listed in Table S2 (Supporting Information). β‐actin was used to normalize sample amplification. The results are shown as relative to the expression level in the corresponding controls, which are assumed as 1.

### Western Blotting

Cells were lysed with RIPA lysis buffer with protease inhibitor and phosphatase inhibitor cocktail (Complete cocktail; Roche Applied Science, Mannheim, Germany). Sample with equal amount of proteins were electrophoresed on sodium dodecyl sulfate polyacrylamide gel before being transferred to polyvinylidene fluoride membrane with 0.45 mM pores (Millipore, Billerica, MA). Membrane was then incubated with primary antibodies followed by secondary antibodies. Antibodies used were listed in Table S1 (Supporting Information). Immunoblotting with anti‐β‐actin antibody was conducted to ensure equal protein loading. Signals were detected using SuperSignal West Femto Maximum Sensitivity Substrate detection system (Thermo Scientific). Images of uncropped blots are shown in Figure S12 (Supporting Information).

### Statistical Analysis

All quantification results were presented as mean ± S.D. (n = 3, unless otherwise indicated). Statistical analysis was performed using two‐tailed unpaired Student's t‐test or log‐rank test conducted using Graph‐Pad Prism 9.0. When more than two groups were compared, one‐way ANOVA analyses were performed. A value of *p* < 0.05 was considered statistically significant.

## Conflict of Interest

The authors declare no conflict of interest.

## Author Contributions

V.K. and S.W. conceptualized and supervised this study; W.D. performed most of the experiments, analyzed, and interpreted the experimental results. R.H, L.W., and C.R. performed part of qRT‐PCR, western blotting, and syngeneic graft mice experiments; R.H., L.W., C.R., F.Z., J.L., V.K., and S.W. analyzed and interpreted the data; W.D., R.H., V.K., and S.W. wrote the paper; H.Z. collected human clinical samples and performed clinical samples analysis; M.M. predicted and constructed shRNA expression vectors.

## Supporting information



Supporting Information

Supplemental Video 1

Supplemental Video 2

Supplemental Video 3

Supplemental Video 4

Supplemental Video 5

Supplemental Video 6

Supplemental Video 7

Supplemental Video 8

Supplemental Video 9

Supplemental Video 10

Supplemental Video 11

Supplemental Video 12

Supplemental Video 13

## Data Availability

The data that support the findings of this study are available from the corresponding author upon reasonable request.
