# Supplementary material for: Chromosome Missegregation Triggers Tumor Cell Pyroptosis and Enhances Anti‐Tumor Immunotherapy in Colorectal Cancer
Source: Adv Sci (Weinh). 2025 Feb 4;12(12):2409769. doi: 10.1002/advs.202409769 (PMC11948012; doi:10.1002/advs.202409769)
Supplement: Supplementary file 1 — Supporting Information [file ADVS-12-2409769-s001.pdf]

ADVANCED SCIENCE
Open Access

Supporting Information

for *Adv. Sci.*, DOI 10.1002/advs.202409769

Chromosome Missegregation Triggers Tumor Cell Pyroptosis and Enhances Anti-Tumor Immunotherapy in Colorectal Cancer

Wei Duan, Rendy Hosea, Lingxian Wang, Cao Ruan, Fuqiang Zhao, Jingyi Liu, Hezhao Zhao, Makoto Miyagishi, Shourong Wu and Vivi Kasim**

Supporting Information for

Chromosome missegregation triggers tumor cell pyroptosis and enhances anti-tumor immunotherapy in colorectal cancer

Wei Duan, Rendy Hosea, Lingxian Wang, Cao Ruan, Fuqiang Zhao, Jingyi Liu, Hezhao Zhao, Makoto Miyagishi, Shourong Wu*, Vivi Kasim*

*E-mail: shourongwu@cqu.edu.cn (S.W.)
vivikasim@cqu.edu.cn (V.K.)

This PDF file includes

Figure S1. *YY2* overexpression induces chromosome missegregation.
Figure S2. *YY2* overexpression suppresses tumorigenesis by enhancing mitotic checkpoint activity.
Figure S3. *YY2* overexpression promotes CTLs activation.
Figure S4. *YY2* overexpression triggers pyroptosis through cytosolic dsDNA-induced AIM2/caspase-1/GSDMD pathway.
Figure S5. Micronuclei envelope instability is crucial for *YY2* overexpression-induced pyroptosis.
Figure S6. Micronuclei envelope instability is important for *YY2* overexpression-induced CTLs activation.
Figure S7. *YY2* knockout fails to trigger micronucleus-mediated pyroptosis.
Figure S8. YY2 is positively correlated with BUB1B.
Figure S9. BUB1B is crucial for *YY2*-induced chromosome missegregation.
Figure S10. BUB1B is crucial for *YY2* overexpresion-induced cytosolic dsDNA response and pyroptosis.
Figure S11. *YY2* overexpression enhances MSS CRC sensitivity to anti-PD-L1 antibody.
Figure S12. Uncropped western blots with the indicated areas of selection in Figs 4, 5, 6, and Supplementary Figs S1, S2, S4, S5, S7, S8, S9.
Table S1. Antibodies used for western blotting, ChIP assay, immunohistochemistry, immunofluorescence, flow cytometry, and *in vivo* PD-L1 blockade.
Table S2. Primer pairs used for qRT-PCR
Videos Legends S1-S13

Other Supplementary Materials for this manuscript include the following:

Videos S1 to S13

Supplementary Figure S1

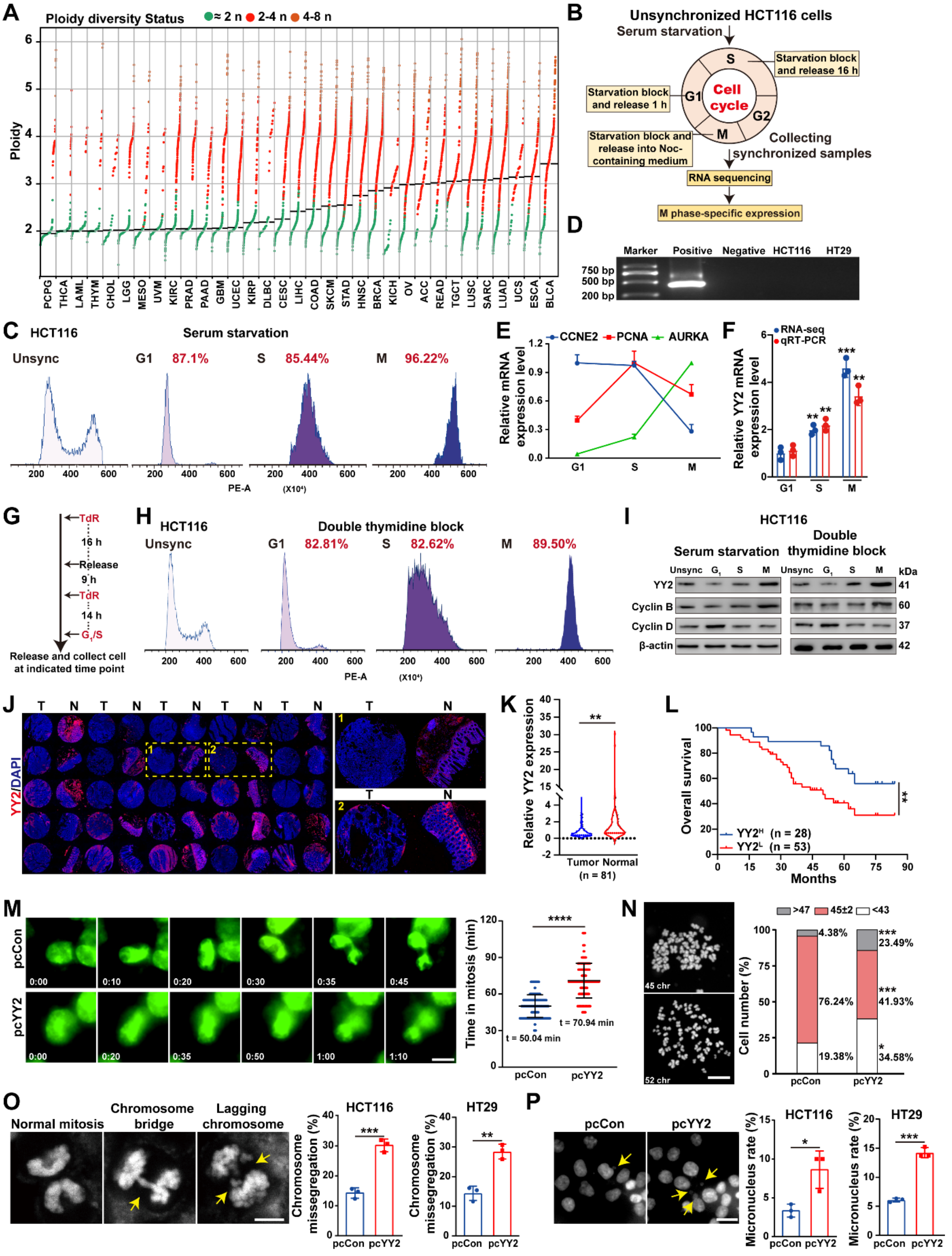

Figure S1. *YY2* overexpression induces chromosome missegregation. A) Ploidy characteristics in different tumor types from TCGA datasets. B) Schematic diagram showing cell cycle synchronization using serum starvation method followed by RNA-seq. C) Percentages of HCT116 cells in each cell

cycle phase synchronized by serum starvation method. Representative images are shown. D) Detection of mycoplasma contamination in HCT116 and HT29 cells. E) Expression profiles of cell cycle phase-specific genes, as obtained from RNA-seq results of synchronized HCT116 cells. F) YY2 expression level in each cell cycle phase, as analyzed using RNA-seq results and qRT-PCR. Data was shown as relative to its expression in G_1 phase. G and H) Cell cycle synchronization using double thymidine block. Schematic diagram (G) and percentages of HCT116 cells in each cell cycle phase (H) are shown. I) YY2 expression level in each cell cycle phase, as examined using western blotting. J and K) YY2 protein level in clinical CRC tissue microarray (n = 81). L) Kaplan-Meier overall survival plot of CRC patients with high ($YY2^H$; n = 28) and low ($YY2^L$; n = 53) YY2 expression levels. M) Mitotic time of *YY2*-overexpressing HCT116 cells. Representative images (scale bars: 20 μm) and scatter plots showing the time-length from NEBD to anaphase (pcCon: total n = 106; pcYY2: total n = 117, pooled from three independent) are shown. N) Chromosome number per cell in *YY2*-overexpressing HCT116 cells. Representative images (scale bars: 10 μm) and quantification results (total cells counted: 50 cells/group) are shown. O) Percentage of cells with chromosome missegregation in *YY2*-overexpressing CRC cells. Representative images (scale bars: 5 μm; *YY2*-overexpressing HCT116 cells) and quantification results (each dot represents one slide, total cell counted >100 mitotic-cells/group) are shown. P) Micronucleus rate in *YY2*-overexpressing CRC cells. Representative images (arrowheads: micronuclei; scale bars: 100 μm; *YY2*-overexpressing HCT116 cells) and micronucleus rate (each dot represents micronucleus rate/slide, total cell counted > 300 cells/slide obtained from 3 independent experiments) are shown. Cells transfected with pcCon were used as control. β-actin was used for qRT-PCR normalization control and as western blotting loading control. Quantification data are shown as mean ± S.D. All data were obtained from three independent experiments, unless otherwise indicated. pcCon: pcEF9-Puro; Unsync: unsynchronized; TdR: thymine deoxyriboside; *$p < 0.05$; **$p < 0.01$; ***$p < 0.001$; ****$p < 0.0001$.

Supplementary Figure S2

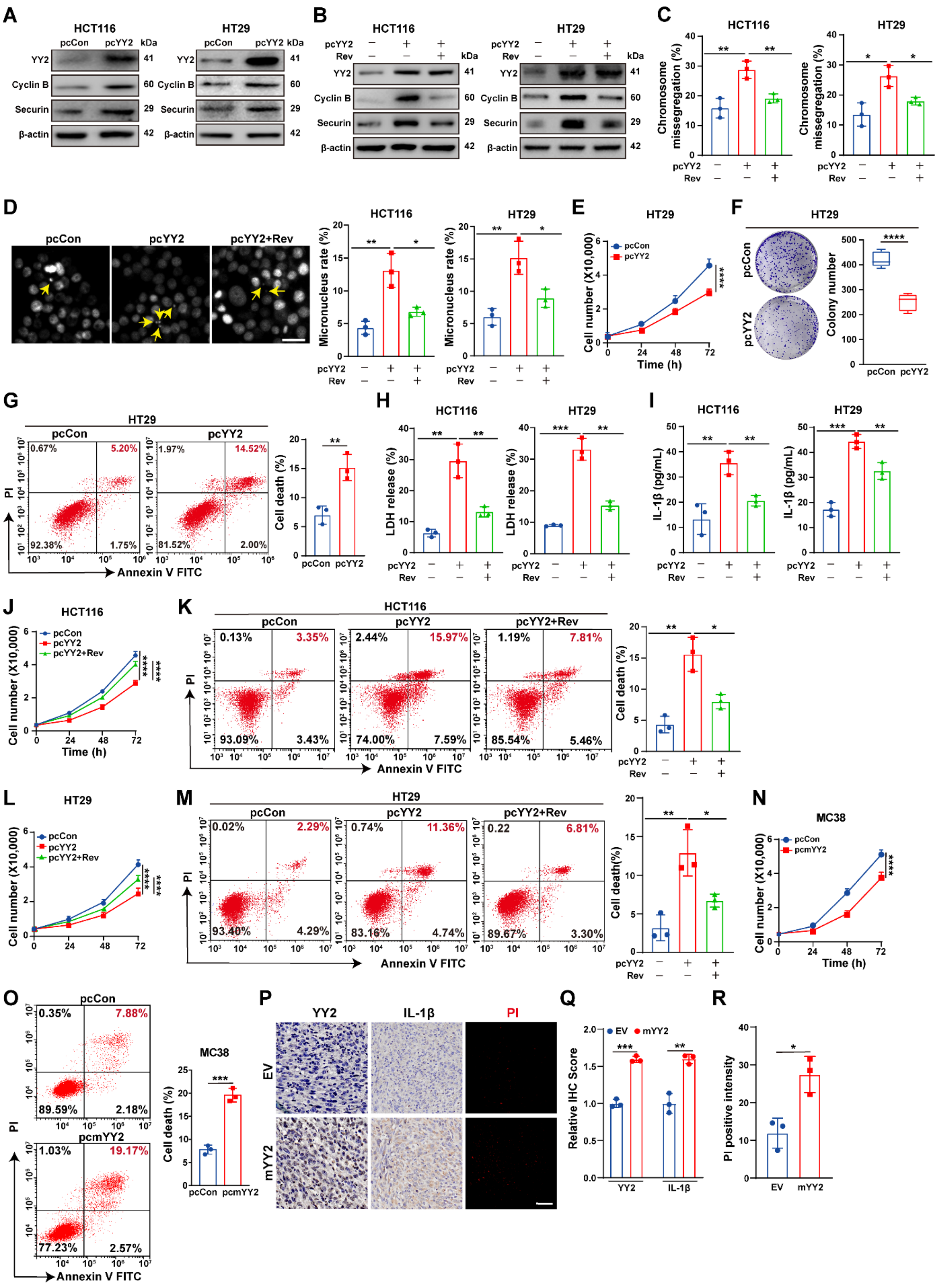

Figure S2. *YY2* overexpression suppresses tumorigenesis by enhancing mitotic checkpoint activity. A) Cyclin B and securin protein levels in *YY2*-overexpressing CRC cells, as examined using western

blotting. B–D) Cyclin B and securin protein levels (B), percentage of cells with chromosome missegregation (C; each dot represents one slide, total cell counted >100 mitotic-cells/group), and micronucleus rate (D; each dot represents micronucleus rate/slide, total cell counted > 300 cells/slide obtained from 3 independent experiments, scale bars: 100 μm) in *YY2*-overexpressing CRC cells treated with reversine (final concentration: 0.2 μM). E and F) Viability at indicated time-points (E) and colony formation potential (F) of *YY2*-overexpressing HT29 cells. G) Cell death rate of *YY2*-overexpressing HT29 cells. H and I) LDH (H) and IL-1β (I) levels released from *YY2*-overexpressing CRC cells treated with reversine (final concentration: 0.2 μM). J–M) Viability at indicated time-points (J, L) and cell death rate (K, M) of *YY2*-overexpressing CRC cells treated with reversine. N and O) Viability at indicated time-points (N) and cell death rate (O) of *YY2*-overexpressing MC38 cells. P–R) YY2 and IL-1β expression levels as well as cell death rate in the syngeneic graft tumor lesions formed by *YY2*-overexpressing MC38 cells, as determined by immunohistochemical staining and *in vivo* PI staining. Representative images (P; scale bars: 200 μm) and quantification results are shown (Q, R; each dot represents quantification results of two slides from the same mice, total 6 slides from 3 mice). Cells transfected with pcCon were used as controls. β-actin was used as western blotting loading control. Quantification data are shown as mean ± S.D. All data were obtained from three independent experiments, unless otherwise indicated. pcCon: pcEF9-Puro; EV: empty lentivirus; Rev: reversine; *$p < 0.05$; **$p < 0.01$; ***$p < 0.001$; ****$p < 0.0001$.

Supplementary Figure S3

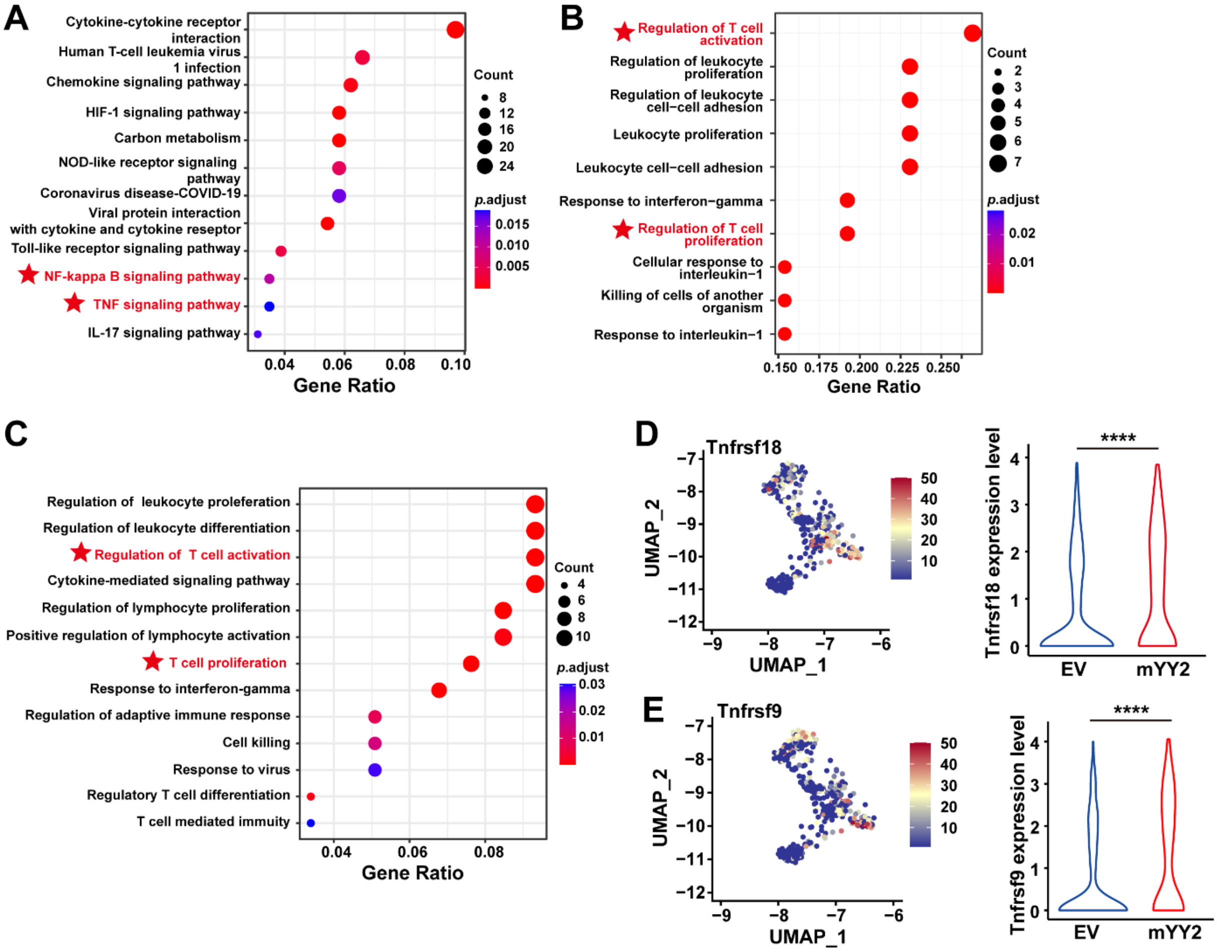

Figure S3. *YY2* overexpression promotes CTLs activation. A–C) KEGG enrichment analysis of upregulated genes in $CD45^+$ cells (A), GO enrichment analysis of upregulated gene in $CD45^+CD11b^-$ cells (B), and KEGG enrichment analysis of $CD45^+CD3^+$ cells (C), as obtained from scRNA-seq results of the syngeneic graft tumor lesions formed by *YY2*-overexpressing MC38 cells. D and E) Tnfrsf18 (D) and Tnfrsf9 (E) expression levels in $CD45^+CD3^+$ T cells from the syngeneic graft tumor lesions formed by *YY2*-overexpressing MC38 cells. Quantification data are shown as mean ± S.D. EV: empty lentivirus; ****$p < 0.0001$.

Supplementary Figure S4

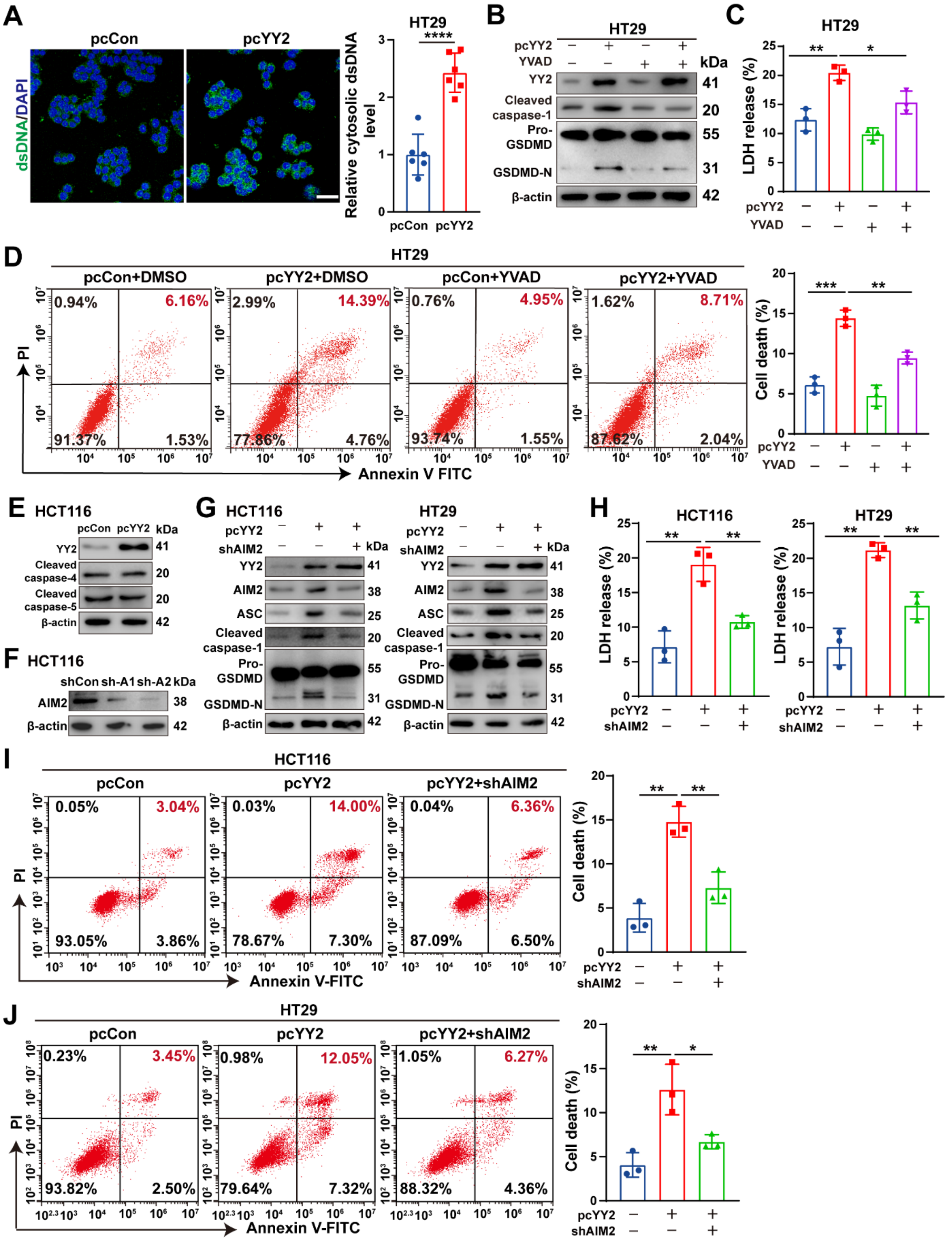

Figure S4. *YY2* overexpression triggers pyroptosis through cytosolic dsDNA-induced AIM2/caspase-1/GSDMD pathway. A) Cytosolic dsDNA in *YY2*-overexpressing HT29 cells (scale bars: 100 μm). B–D) Expression levels of AIM2 pathway-related proteins (B), LDH release (C) from and cell death rate

(D) in *YY2*-overexpressing HT29 cells treated with Z-YVAD-FMK (final concentration: 10 μM) for 48 h. E) Cleaved caspases-4 and 5 expression levels in *YY2*-overexpressing HCT116 cells. F) AIM2 protein expression level in HCT116 cells transfected with shRNA expression vectors targeting *AIM2*. G–J) Expression levels of AIM2 pathway-related proteins (G), LDH release (H) from and cell death rate (I, J) in *AIM2*-knockdown, *YY2*-overexpressing HCT116 and HT29 cells. Cells transfected with pcCon or shCon were used as controls. β-actin was used as western blotting loading control. Quantification data are shown as mean ± S.D. All data were obtained from three independent experiments, unless otherwise indicated. pcCon: pcEF9-Puro; YVAD: Z-YVAD-FMK; *$p < 0.05$; **$p < 0.01$; ***$p < 0.001$; ****$p < 0.0001$.

Supplementary Figure S5

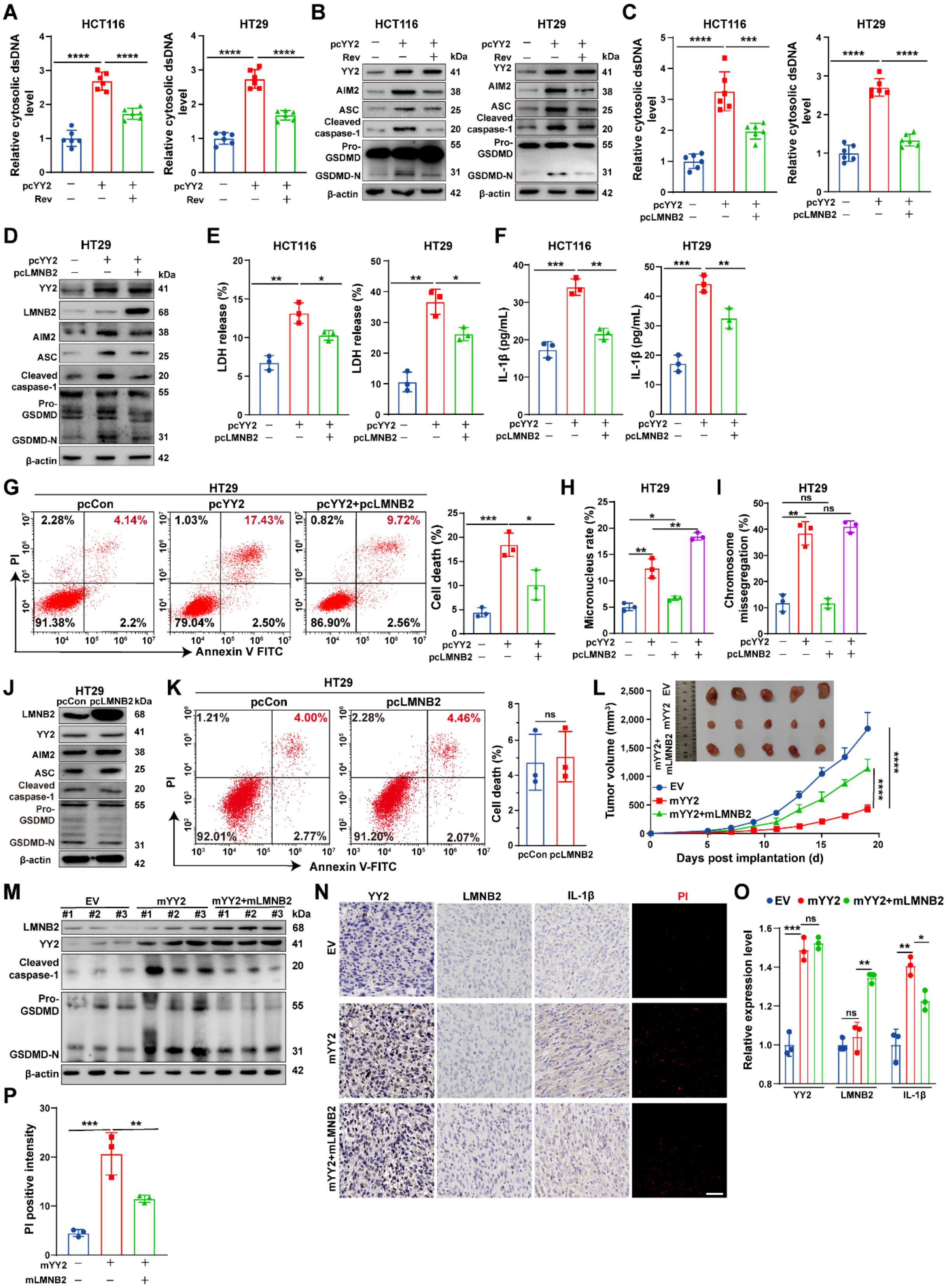

Figure S5. Micronuclei envelope instability is crucial for *YY2* overexpression-induced pyroptosis. A and B) Cytosolic dsDNA (A) and expression levels of AIM2 pathway-related proteins (B) in *YY2*-

overexpressing CRC cells treated with reversine (final concentration: 0.2 μM). C) Cytosolic dsDNA in *LMNB2*-overexpressing, *YY2*-overexpressing CRC cells. D–G) Expression levels of AIM2 pathway-related proteins (D), LDH (E), IL-1β levels released from (F) and cell death rate in (G) *LMNB2*-overexpressing, *YY2*-overexpressing CRC cells. H) Micronucleus rate in *LMNB2*-overexpressing, *YY2*-overexpressing CRC cells (each dot represents micronucleus rate/slide, total cell counted > 300 cells/slide obtained from 3 independent experiments). I) Percentage of cells with chromosome missegregation in *LMNB2*-overexpressing, *YY2*-overexpressing HT29 cells (each dot represents one slide, total cell counted >100 mitotic-cells/group). J and K) Expression levels of AIM2 pathway-related proteins (J) and cell death rate (K) in *LMNB2*-overexpressing HT29 cells. L) Tumor volume and morphological images of syngeneic graft tumors formed by *LMNB2*-overexpressing, *YY2*-overexpressing MC38 cells (n = 5/group). M) Expression levels of AIM2 pathway-related proteins in the syngeneic graft tumor lesions formed by *LMNB2*-overexpressing, *YY2*-overexpressing MC38 cells. N–P) YY2 and IL-1β expression levels as well as cell death rate in the syngeneic graft tumor lesions formed by *LMNB2*-overexpressing, *YY2*-overexpressing MC38 cells, as determined by immunohistochemical staining and *in vivo* PI staining. Representative images (N; scale bars: 200 μm) and quantification results (O, P; each dot represents quantification results of two slides from the same mice, total 6 slides from 3 mice) are shown. Cells transfected with pcCon were used as controls. β-actin was used as western blotting loading control. Quantification data are shown as mean ± S.D. All data were obtained from three independent experiments, unless otherwise indicated. pcCon: pcEF9-Puro; EV: empty lentivirus; Rev: reversine; ns: not significant; *$p < 0.05$; **$p < 0.01$; ***$p < 0.001$; ****$p < 0.0001$.

Supplementary Figure S6

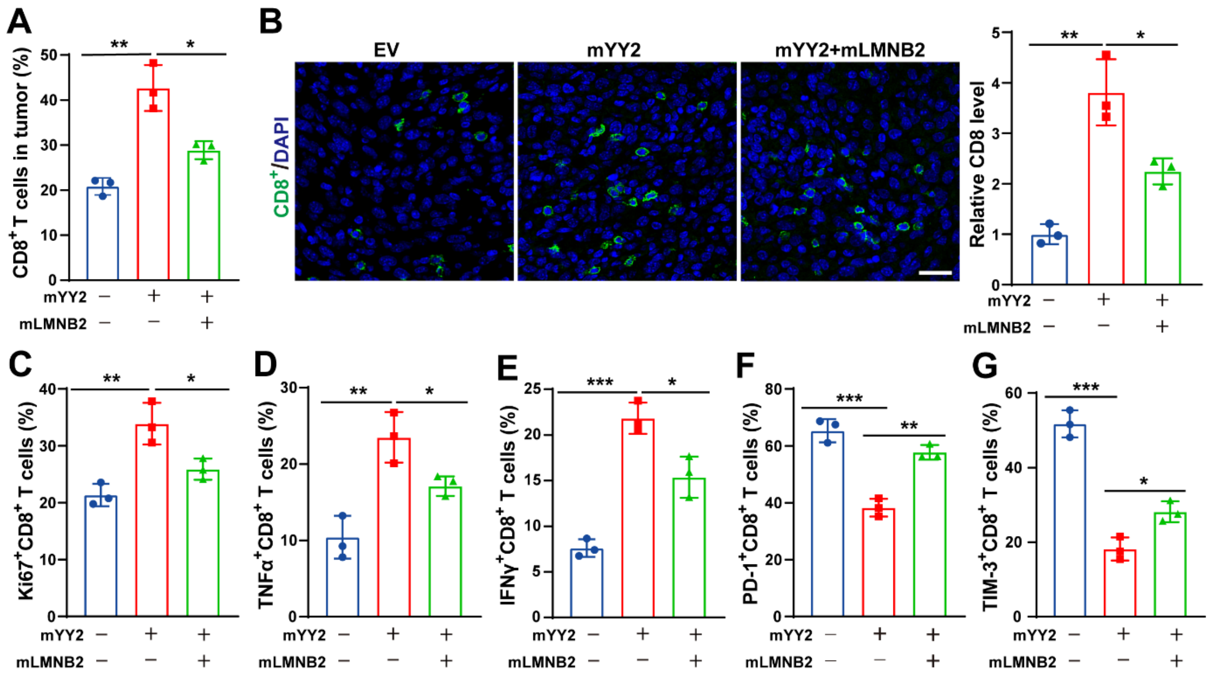

Figure S6. Micronuclei envelope instability is important for *YY2* overexpression-induced CTLs activation. A and B) Percentages of CD8$^+$ T cells in the syngeneic graft tumor lesions formed by *LMNB2*-overexpressing, *YY2*-overexpressing MC38 cells, as examined using flow cytometry (A; three tumors from three mice for each group) and immunofluorescent staining (B; left panels: representative images, scale bars: 100 μm; right panels: quantification results, each dot represents quantification results of two slides from the same mice, total 6 slides from 3 mice). C–G) Percentages of Ki67$^+$CD8$^+$ (C), TNFα$^+$ CD8$^+$ (D), IFNγ$^+$CD8$^+$ (E), PD-1$^+$CD8$^+$ (F), and TIM-3$^+$CD8$^+$ (G) T cells in the syngeneic graft tumor lesions formed by *LMNB2*-overexpressing, *YY2*-overexpressing MC38 cells (three tumors from three mice for each group). Quantification data are shown as mean ± S.D. EV: empty lentivirus; *p < 0.05; **p < 0.01; ***p < 0.001.

Supplementary Figure S7

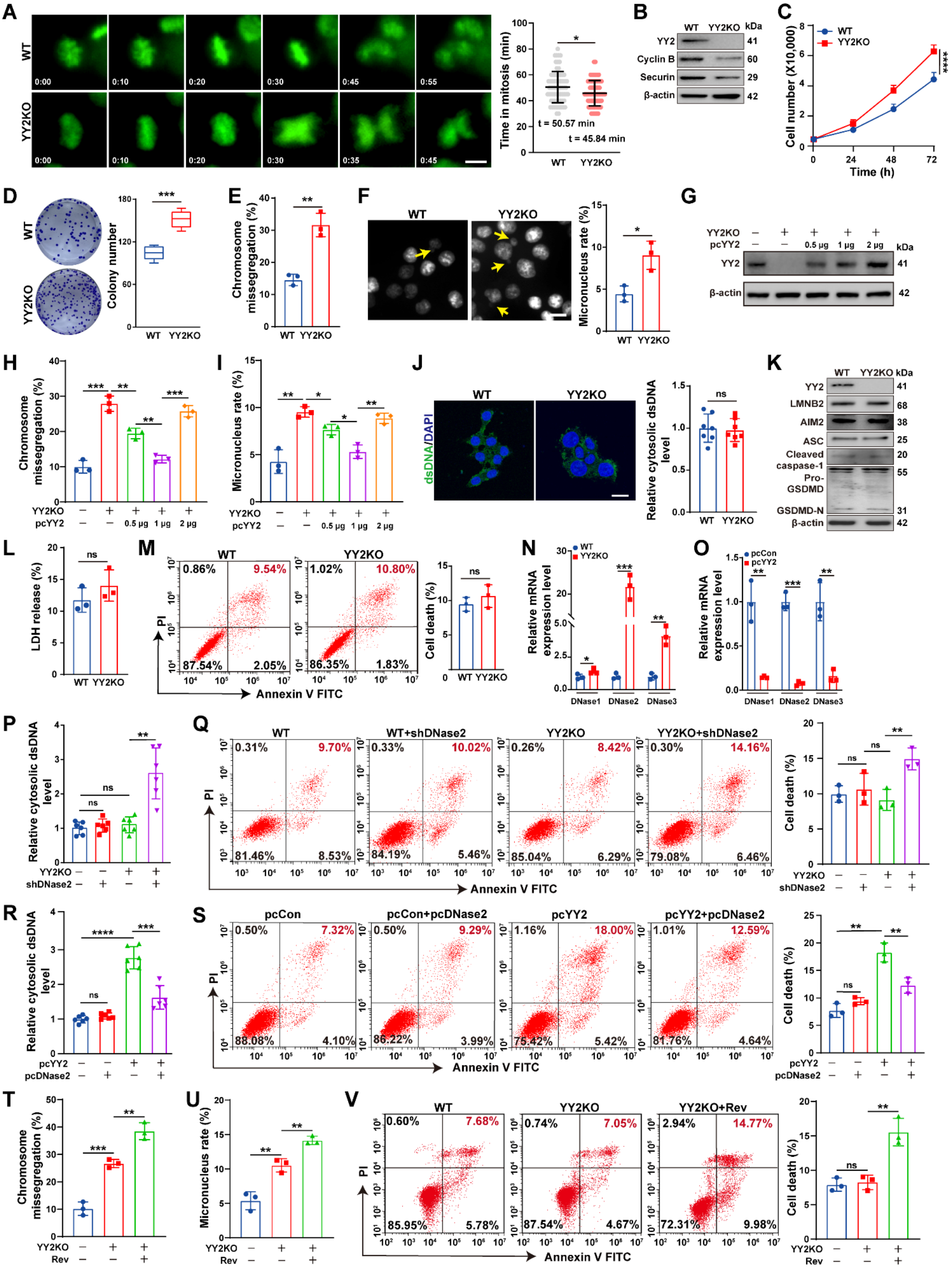

Figure S7. *YY2* knockout fails to trigger micronucleus-mediated pyroptosis. A) Mitotic time of

HCT116^{YY2KO} cells. Representative images (scale bars: 20 μm) and scatter plots showing the time-length from NEBD to anaphase (WT: total n = 70; YY2KO: total n = 71, pooled from three independent experiments) are shown. B) Cyclin B and securin protein levels in HCT116^{YY2KO} cells. C and D) Viability at indicated time-points (C) and colony formation potential (D) of HCT116^{YY2KO} cells. E and F) Percentage of cells with chromosome missegregation (E; each dot represents one slide, total cell counted > 100 mitotic-cells/group) and micronucleus rate (F; each dot represents micronucleus rate/slide, total cell counted > 300 cells/slide obtained from 3 independent experiments, scale bars: 100 μm) in HCT116^{YY2KO} cells. G) YY2 protein expression level in HCT116^{YY2KO} cells transfected with indicated amount of *YY2* overexpression vectors, as determined using western blotting. H and I) Percentage of cells with chromosome missegregation (H) and micronucleus rate (I) in HCT116^{YY2KO} cells transfected with indicated amount of *YY2* overexpression vectors. J and K) Cytosolic dsDNA (J; scale bars: 100 μm), and expression levels of LMNB2 and AIM2 pathway-related proteins (K) in HCT116^{YY2KO} cells. L and M) LDH release (L) from and cell death rate (M) in HCT116^{YY2KO} cells. N and O) mRNA expression levels of DNase family in HCT116^{YY2KO} (N) and *YY2*-overexpressing (O) HCT116 cells. P and Q) Cytosolic dsDNA (P) and cell death rate (Q) in *DNase2*-knocked down HCT116^{YY2KO} cells. R and S) Cytosolic dsDNA (R) and cell death rate (S) in *DNase2*-overexpressed *YY2*-overexpressing HCT116 cells. T–V) Percentage of cells with chromosome missegregation (T) and micronucleus rate (U), as well as cell death rate (V) in HCT116^{YY2KO} cells treated with reversine (final concentration: 0.2 μM). Cells transfected with pcCon, shCon or wild-type HCT116 cells were used as controls. β-actin was used for qRT-PCR normalization and as western blotting loading control. Quantification data are shown as mean ± S.D. All data were obtained from three independent experiments, unless otherwise indicated. pcCon: pcEF9-Puro; Rev: reversine; ns: not significant; *p < 0.05; **p < 0.01; ***p < 0.001; ****p < 0.0001.

Supplementary Figure S8

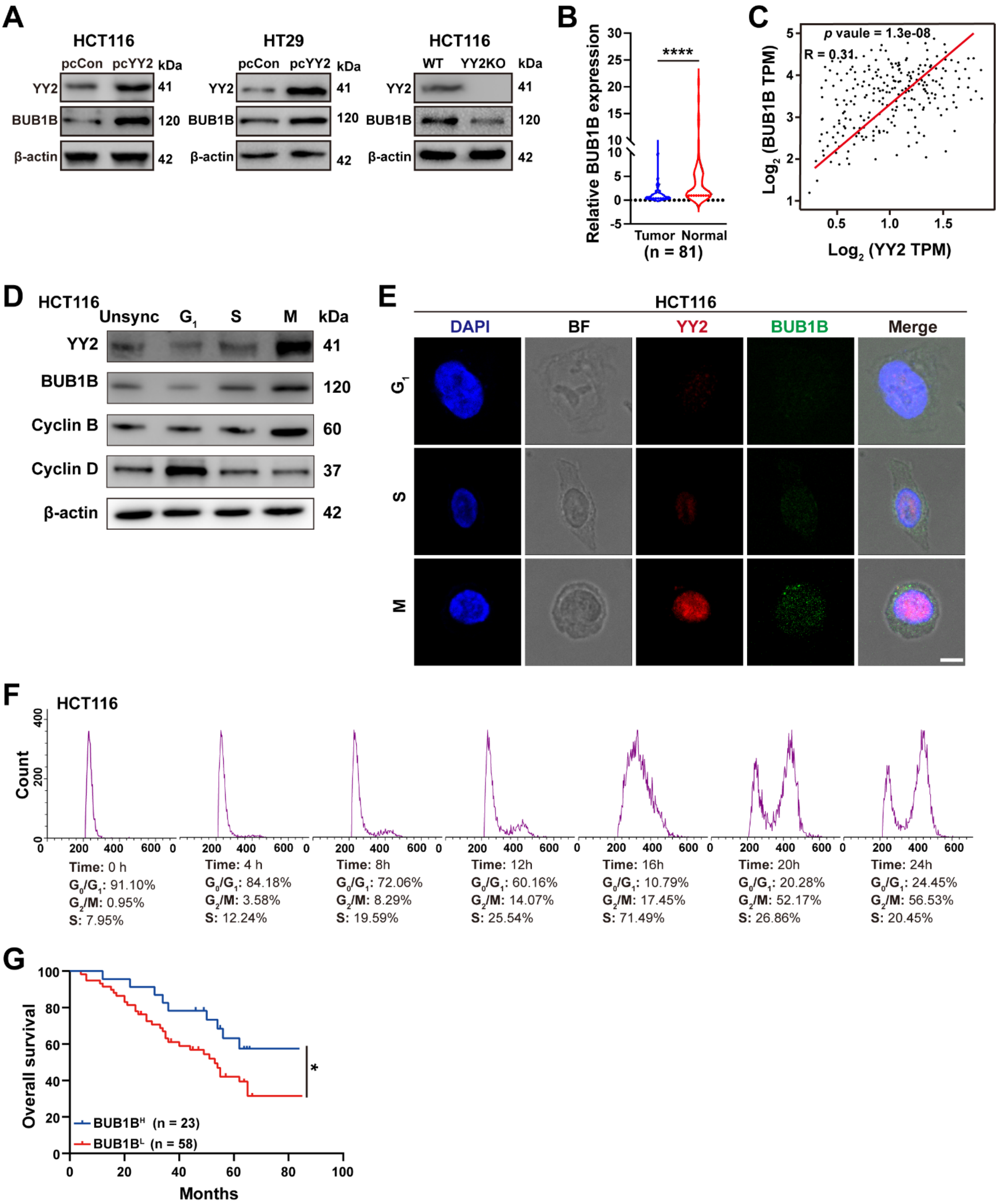

Figure S8. YY2 is positively correlated with BUB1B. A) BUB1B protein level in *YY2*-overexpressing CRC cells and HCT116^{YY2KO} cells. B) BUB1B protein level in clinical CRC tissue microarray (n = 81). C) Correlation of YY2 and BUB1B expression in clinical CRC tissues. D and E) BUB1B and YY2 protein expression levels in HCT116 cells at indicated cell phase by western blot (D) and

immunofluorescence staining (E; scale bars: 50 μm). F) HCT116 cell cycle progression at indicated time-points starting immediately after serum starvation release. G) Kaplan-Meier overall survival plot of CRC patients with high ($BUB1B^H$; n = 23) and low ($BUB1B^L$; n = 58) BUB1B expression level. Cells transfected with pcCon or wild-type cells were used as controls. β-actin was used for western blotting loading control. Quantification data are shown as mean ± S.D. All data were obtained from three independent experiments, unless otherwise indicated. Unsync: unsynchronized; pcCon: pcEF9-Puro; *$p < 0.05$; ****$p < 0.0001$.

Supplementary Figure S9

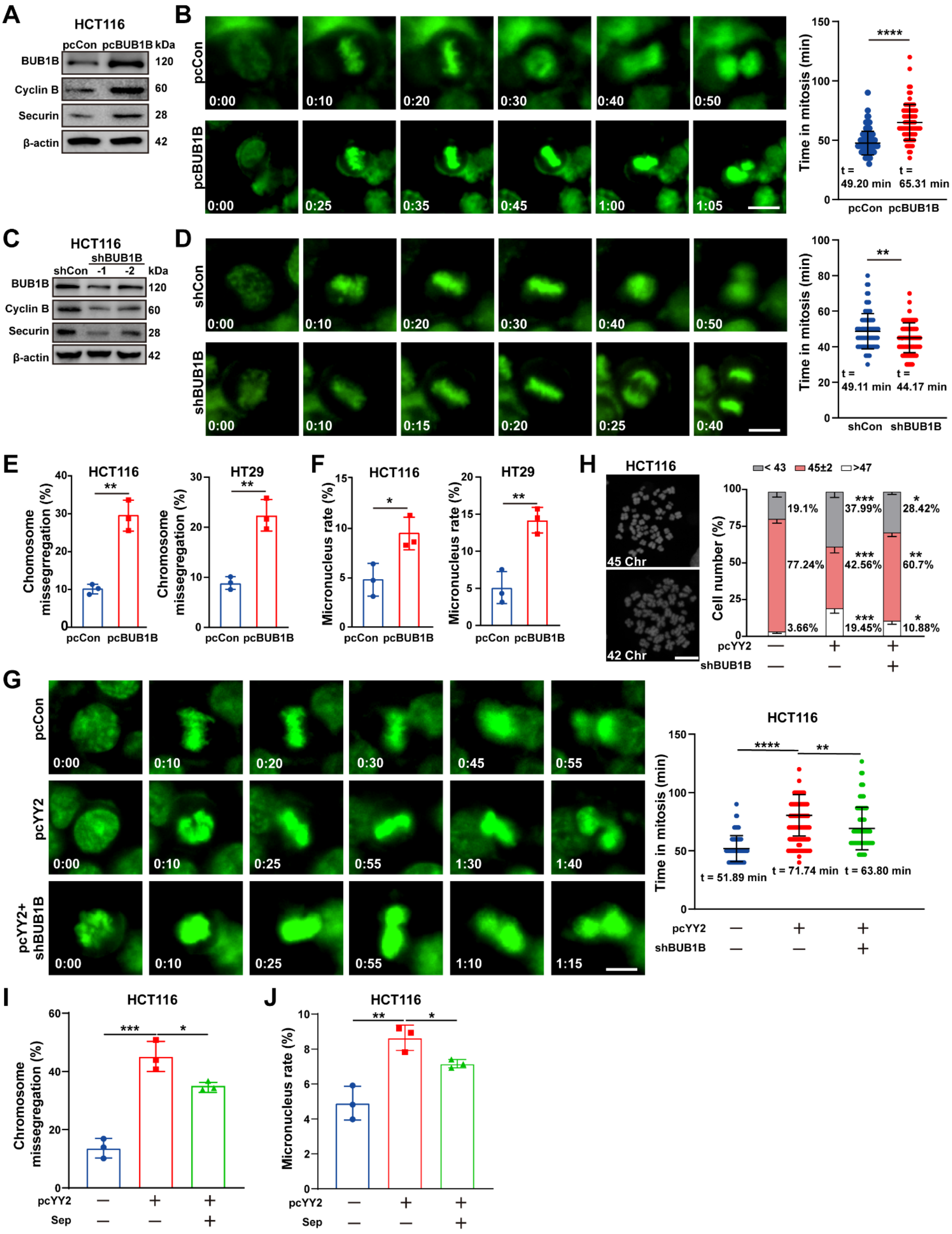

Figure S9. BUB1B is crucial for *YY2*-induced chromosome missegregation. A) Cyclin B and securin protein levels in *BUB1B*-overexpressing HCT116 cells. B) Mitotic time of *BUB1B*-overexpressing

HCT116 cells (pcCon: total n = 87; pcBUB1B: total n =81, pooled from three independent experiments). C) Cyclin B and securin protein levels in *BUB1B*-knocked down HCT116 cells. shBUB1B-1 was used in subsequent experiments. D) Mitotic time of *BUB1B*-knocked down HCT116 cells (shCon: total n = 79; shBUB1B: total n =84, pooled from three independent experiments). E and F) Percentage of cells with chromosome missegregation (E; each dot represents one slide, total cell counted > 100 mitotic-cells/group) and micronucleus rate (F; each dot represents micronucleus rate/slide, total cell counted > 300 cells/slide obtained from 3 independent experiments, scale bars: 100 μm) in *BUB1B*-overexpressing CRC cells. G) Mitotic time of *BUB1B*-knocked down, *YY2*-overexpressing HCT116 cells (pcCon: total n = 87; pcYY2: total n =81; pcYY2+shBUB1B: total n = 121, pooled from three independent experiments). H) Chromosome number per cell in *BUB1B*-knocked down, *YY2*-overexpressing HCT116 cells. Representative images (scale bars: 10 μm) and quantification results (total cells counted: 50 cells/group) are shown. I and J) Percentage of cells with chromosome missegregation (I) and micronucleus rate (J) in *YY2*-overexpressing HCT116 cells treated with sepantronium bromide (final concentration: 1 μM) for 24 h. Representative images (scale bars: 20 μm) and scatter plots showing the time-length from NEBD to anaphase are shown. Cells transfected with pcCon or shCon were used as controls. β-actin was used for western blotting loading control. Quantification data are shown as mean ± S.D. All data were obtained from three independent experiments, unless otherwise indicated. pcCon: pcEF9-Puro; Sep: sepantronium bromide; *$p < 0.05$; **$p < 0.01$; ***$p < 0.001$; ****$p < 0.0001$.

Supplementary Figure S10

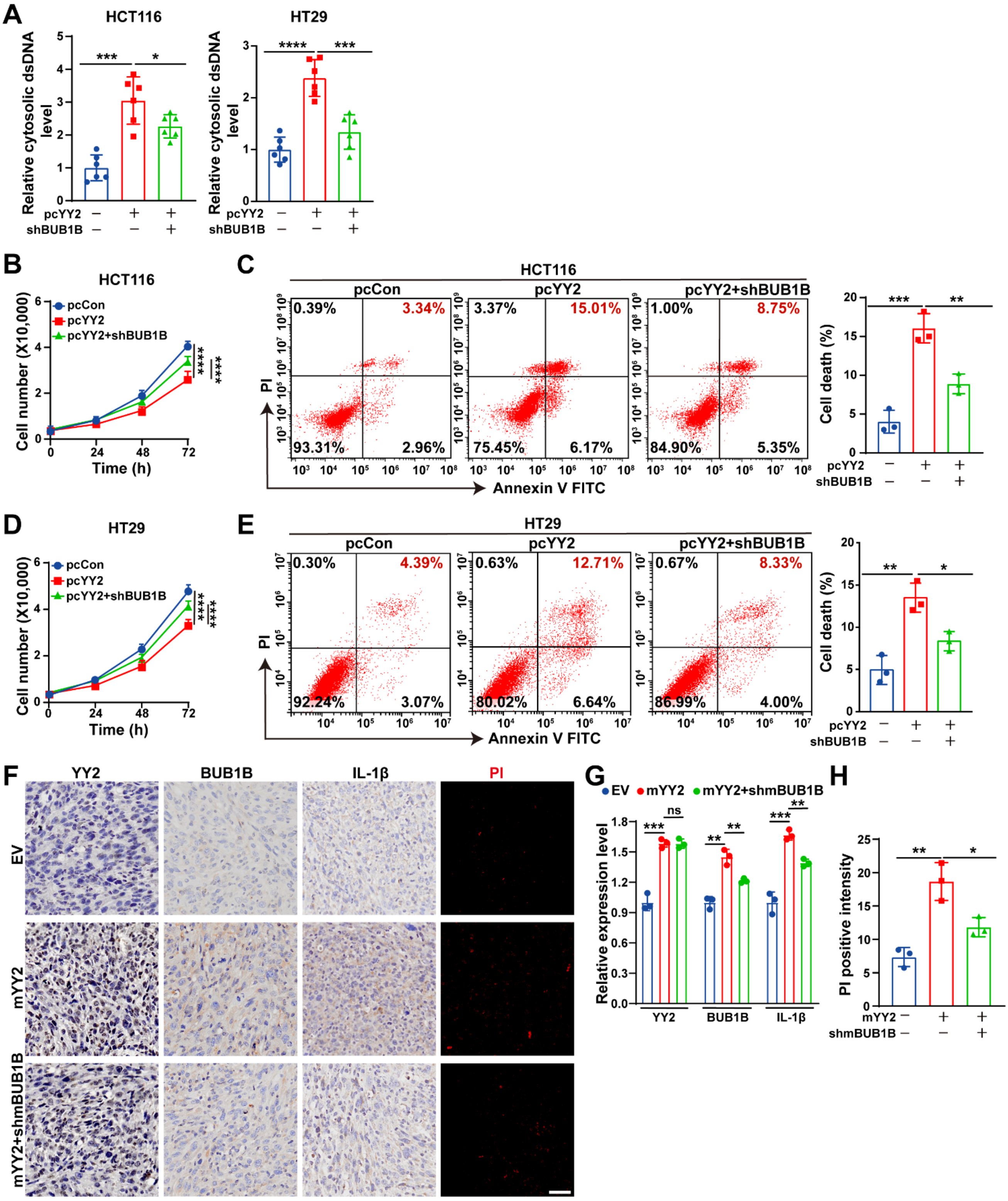

Figure S10. BUB1B is crucial for *YY2* overexpression-induced cytosolic dsDNA response and pyroptosis. A) Cytosolic dsDNA in *BUB1B*-knocked down, *YY2*-overexpressing CRC cells. B–E) Viability at indicated time-points (B, D) and cell death rate (C, E) of *BUB1B*-knocked down, *YY2*-overexpressing CRC cells. F–H) YY2 and IL-1β expression levels, as well as cell death rate in the

syngeneic graft tumor lesions formed by *BUB1B*-knocked down, *YY2*-overexpressing MC38 cells, as determined by immunohistochemical staining and *in vivo* PI staining. Representative images (F; scale bars: 200 μm) and quantification results (G, H, each dot represents quantification results of two slides from the same mice, total 6 slides from 3 mice) are shown. Cells transfected with pcCon and shCon were used as controls. Quantification data are shown as mean ± S.D. All data were obtained from three independent experiments, unless otherwise indicated. pcCon: pcEF9-Puro; EV: empty lentivirus; ns: not significant; *$p < 0.05$; **$p < 0.01$; ***$p < 0.001$; ****$p < 0.0001$.

Supplementary Figure S11

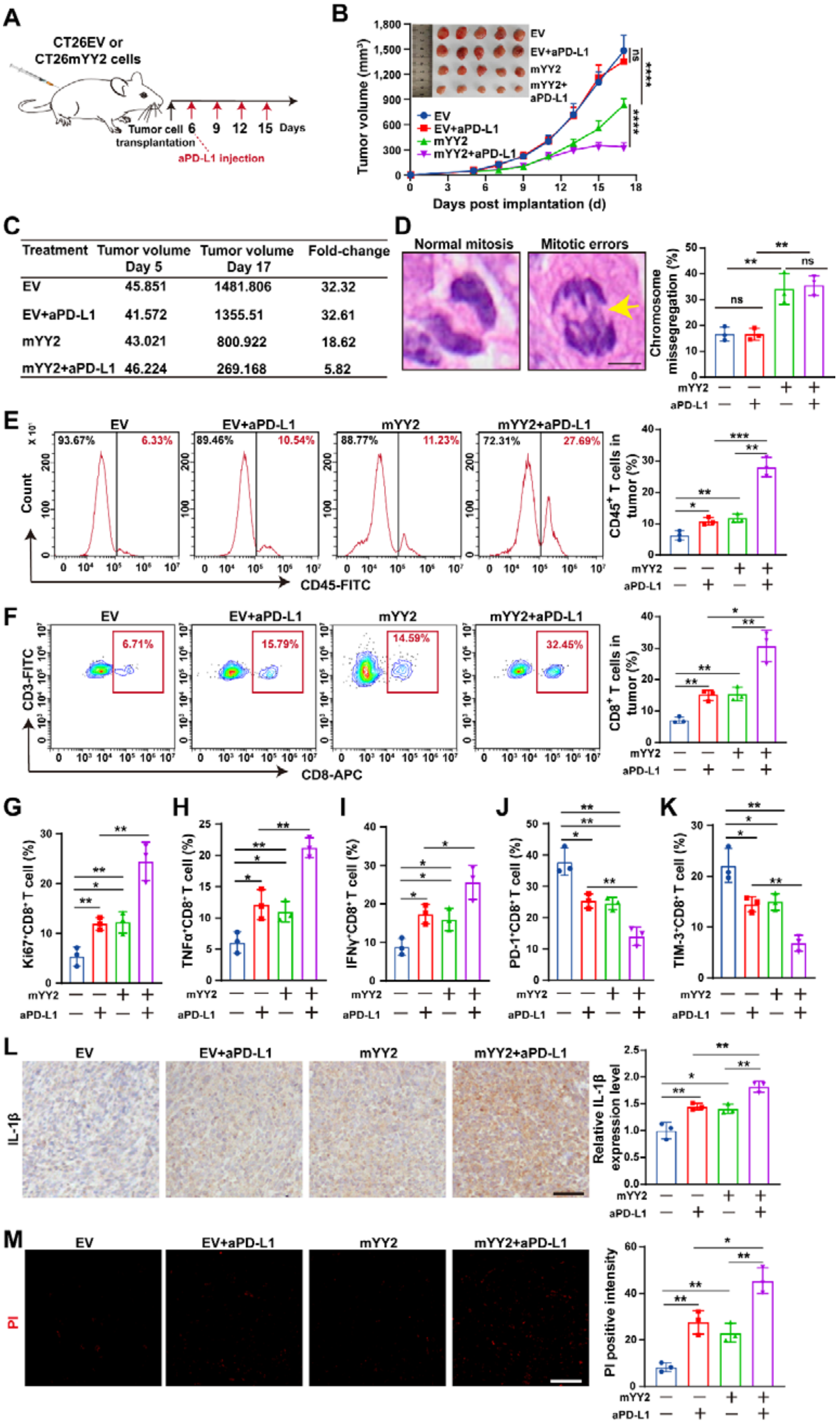

Figure S11. *YY2* overexpression enhances MSS CRC sensitivity to anti-PD-L1 antibody. A) Schematic diagram showing the combinatorial therapy procedure in MSS CRC mouse model. B) Tumor volume

and morphological images of the syngeneic graft tumors formed by *YY2*-overexpressing CT26 cells and treated with anti-PD-L1 antibody at indicated time-points (final concentration: 10 mg/kg bodyweight; n = 5/group). C) Fold-change of syngeneic graft tumor volumes at day 17 compared to those at the starting point of treatment (day 5). D) Percentage of cells with chromosome missegregation in the syngeneic graft tumor lesions formed by *YY2*-overexpressing CT26 cells and treated with anti-PD-L1 antibody. Representative images of chromosome missegregation (indicated by arrowheads, scale bars: 5 μm) and quantification results (each dot represents one slide, total cell counted > 100 mitotic-cells/group) are shown. E and F) Percentages of $CD45^+$ cells (E) and $CD8^+$ T cells (F) in syngeneic graft tumor lesions formed by *YY2*-overexpressing CT26 cells (three tumors from three mice for each group). G–K) Percentages of $Ki67^+CD8^+$ (G), $TNF\alpha^+CD8^+$ (H), $IFN\gamma^+CD8^+$ (I), $PD\text{-}1^+CD8^+$ (J), and $TIM\text{-}3^+CD8^+$ (K) T cells in the syngeneic graft tumor lesions formed by *YY2*-overexpressing CT26 cells and treated with anti-PD-L1 antibody (three tumors from three mice for each group). L and M) IL-1β expression level (L) and cell death rate (M) in the syngeneic graft tumor lesions formed by *YY2*-overexpressing CT26 cells and treated with anti-PD-L1 antibody, as determined by immunohistochemical staining and *in vivo* PI staining. Representative images (left panels; scale bars: 200 μm) and quantification results (right panels; each dot represents quantification results of two slides from the same mice, total 6 slides from 3 mice) are shown. Quantification data are shown as mean ± S.D. EV: empty lentivirus; ns: not significant; *$p < 0.05$; **$p < 0.01$; ***$p < 0.001$; ****$p < 0.0001$.

Supplementary Figure S12

A

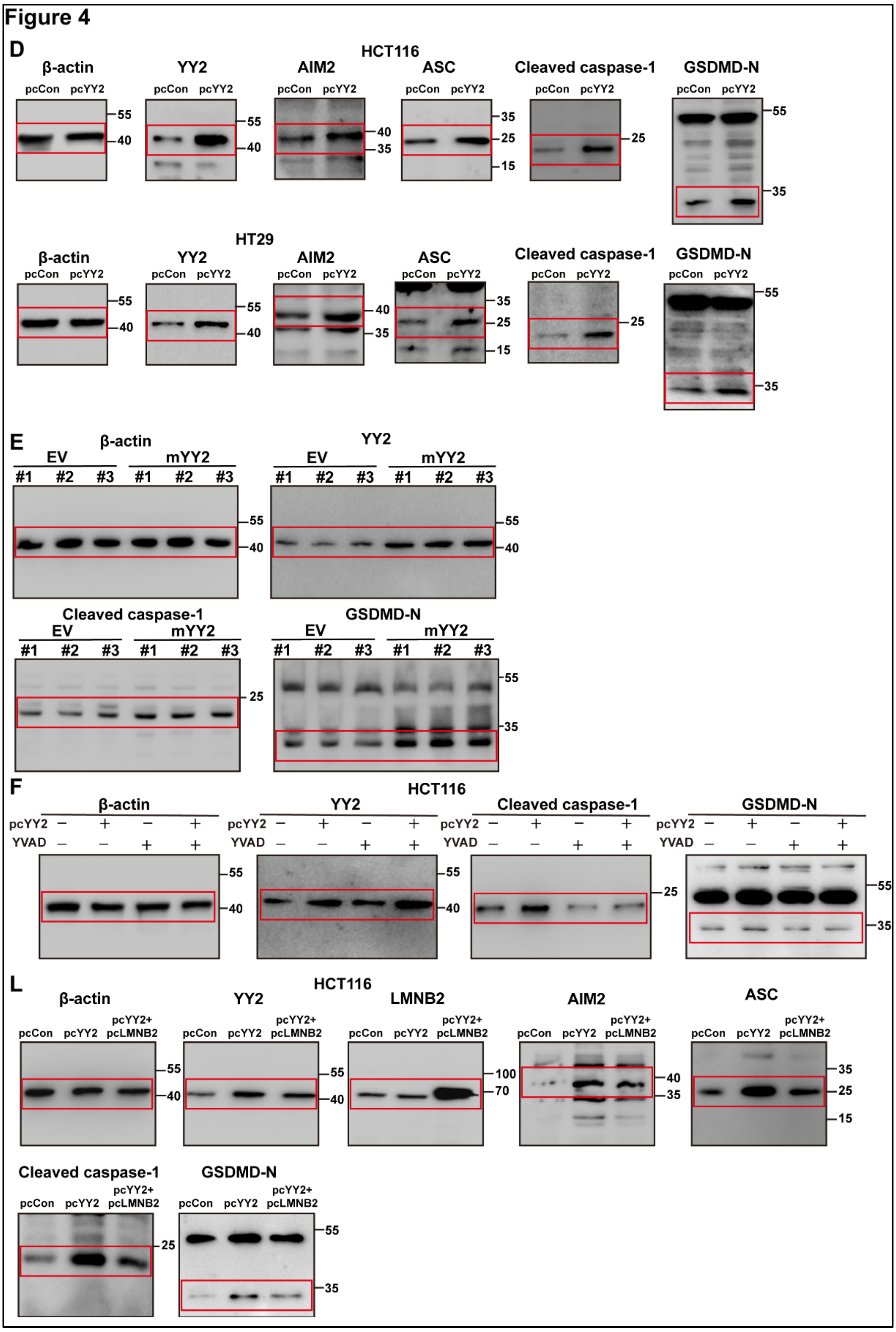

Figure S12. Uncropped western blots with the indicated areas of selection in Figs 4, 5, 6 and Supplementary Figs S1, S2, S4, S5, S7, S8 and S9. (continued)

B

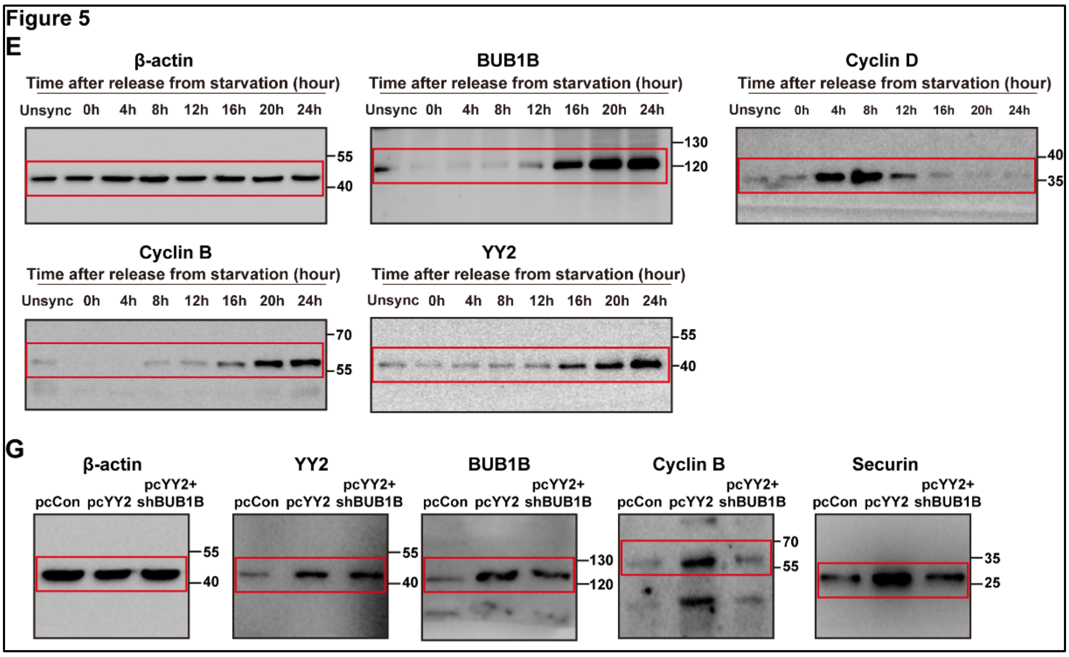

Figure S12. Uncropped western blots with the indicated areas of selection in Figs 4, 5, 6 and Supplementary Figs S1, S2, S4, S5, S7, S8 and S9. (continued)

C

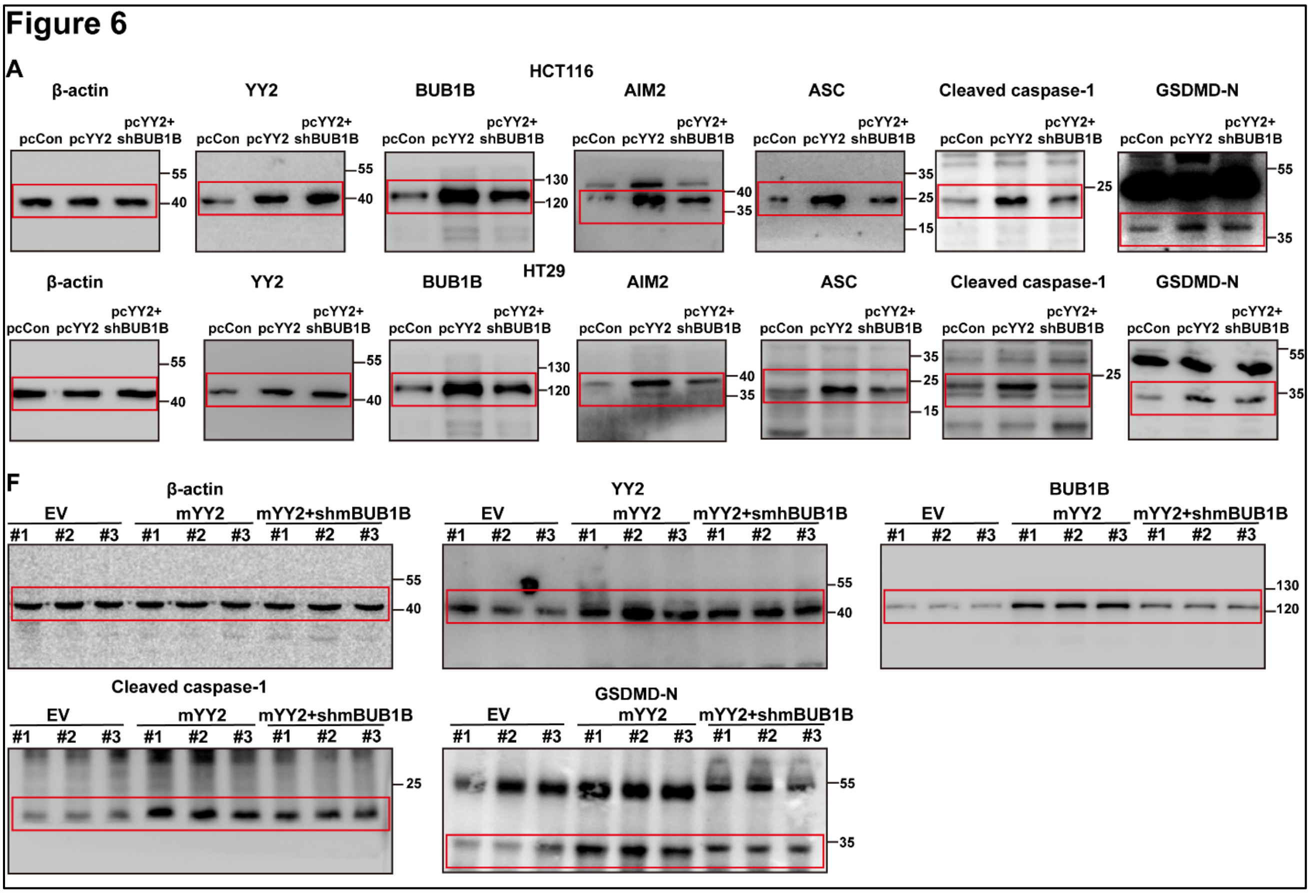

Figure S12. Uncropped western blots with the indicated areas of selection in Figs 4, 5, 6 and Supplementary Figs S1, S2, S4, S5, S7, S8 and S9. (continued)

D

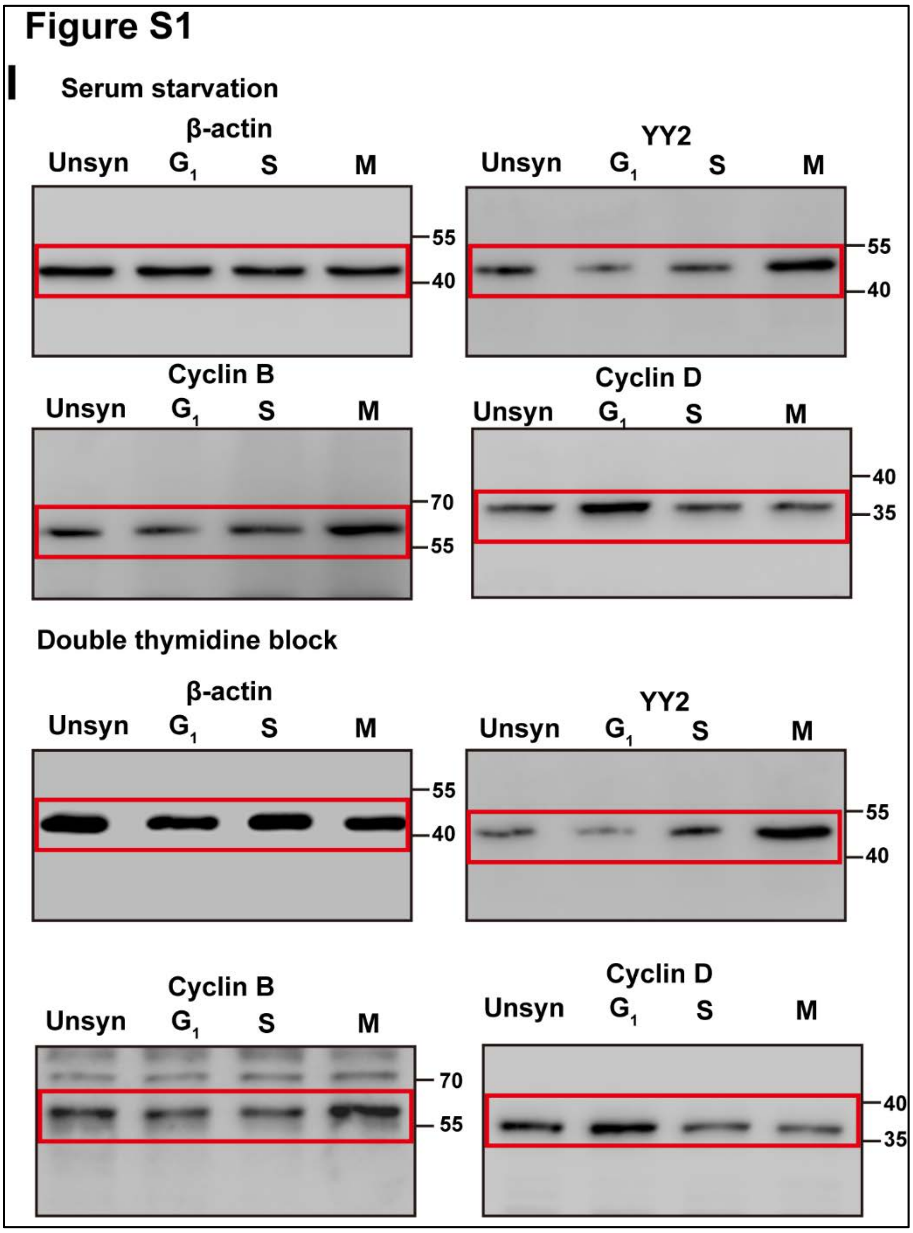

Figure S12. Uncropped western blots with the indicated areas of selection in Figs 4, 5, 6 and Supplementary Figs S1, S2, S4, S5, S7, S8 and S9. (continued)

E

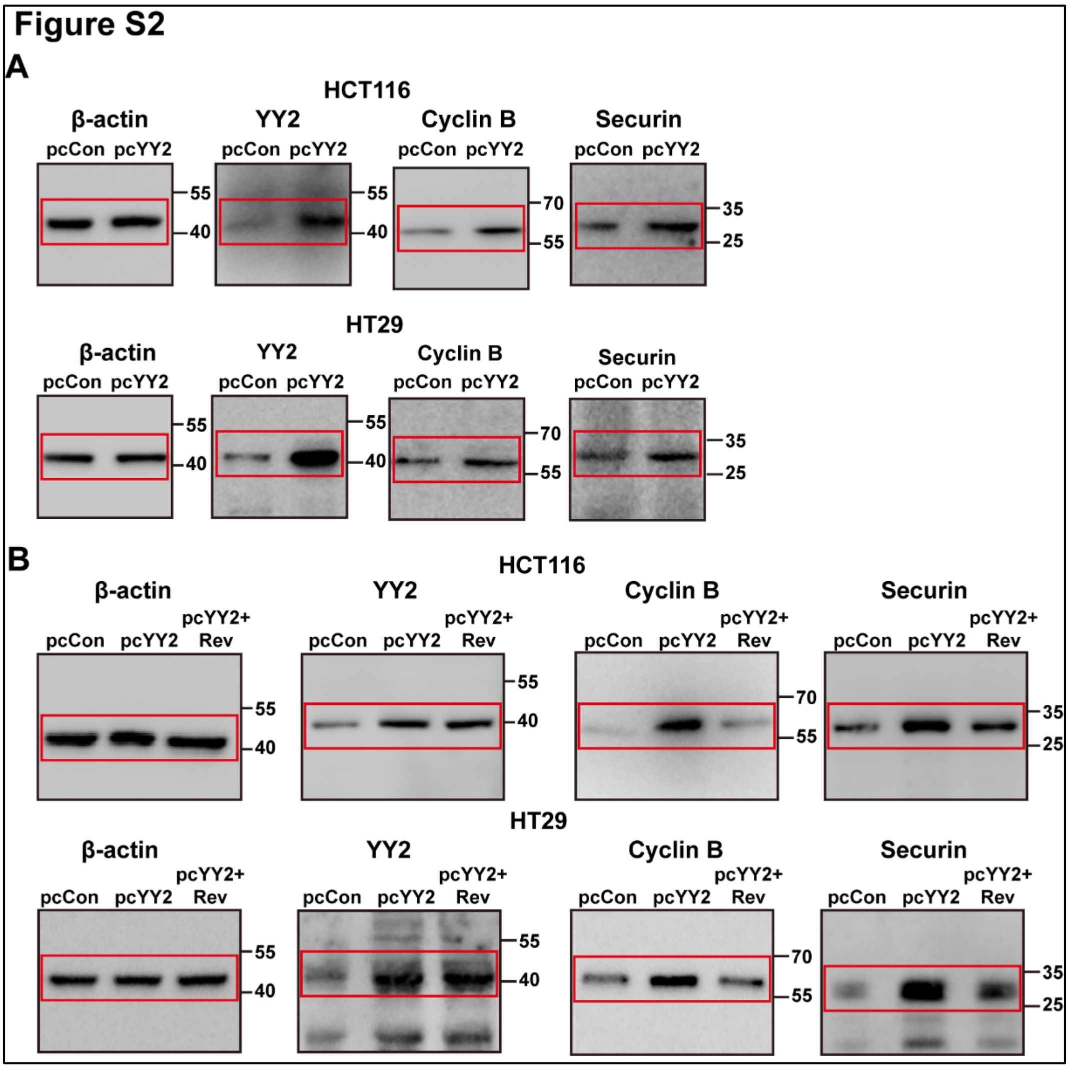

Figure S12. Uncropped western blots with the indicated areas of selection in Figs 4, 5, 6 and Supplementary Figs S1, S2, S4, S5, S7, S8 and S9. (continued)

F

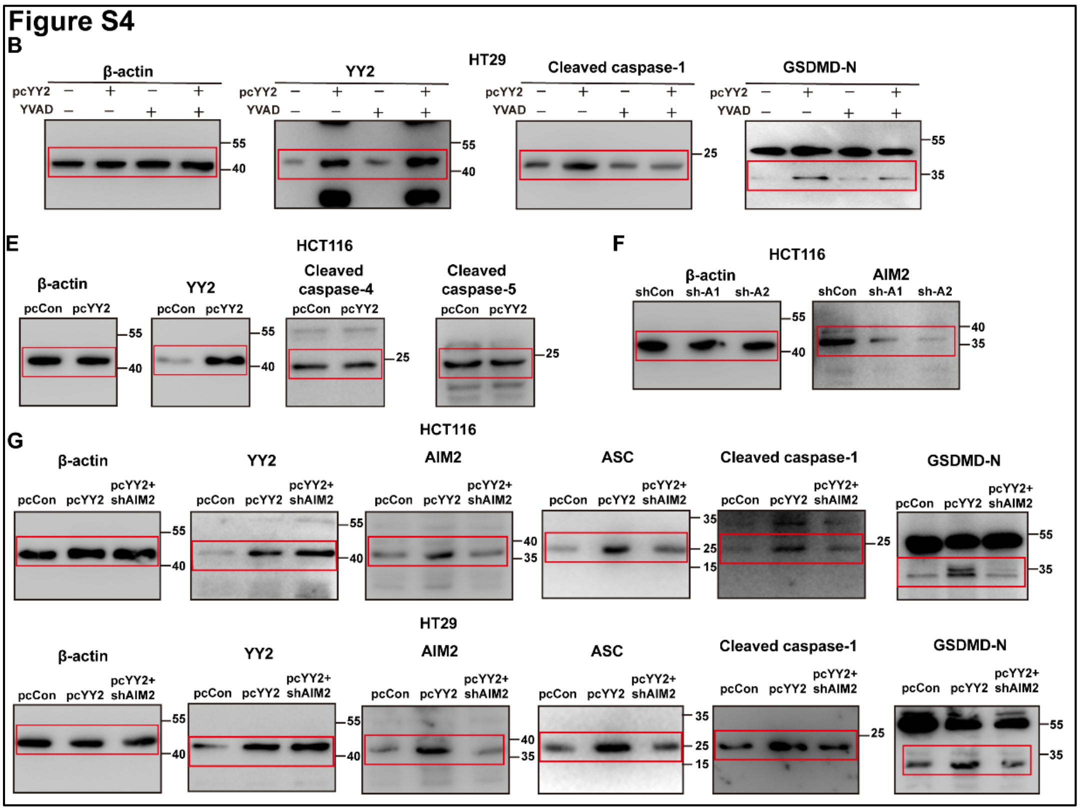

Figure S12. Uncropped western blots with the indicated areas of selection in Figs 4, 5, 6 and Supplementary Figs S1, S2, S4, S5, S7, S8 and S9. (continued)

G

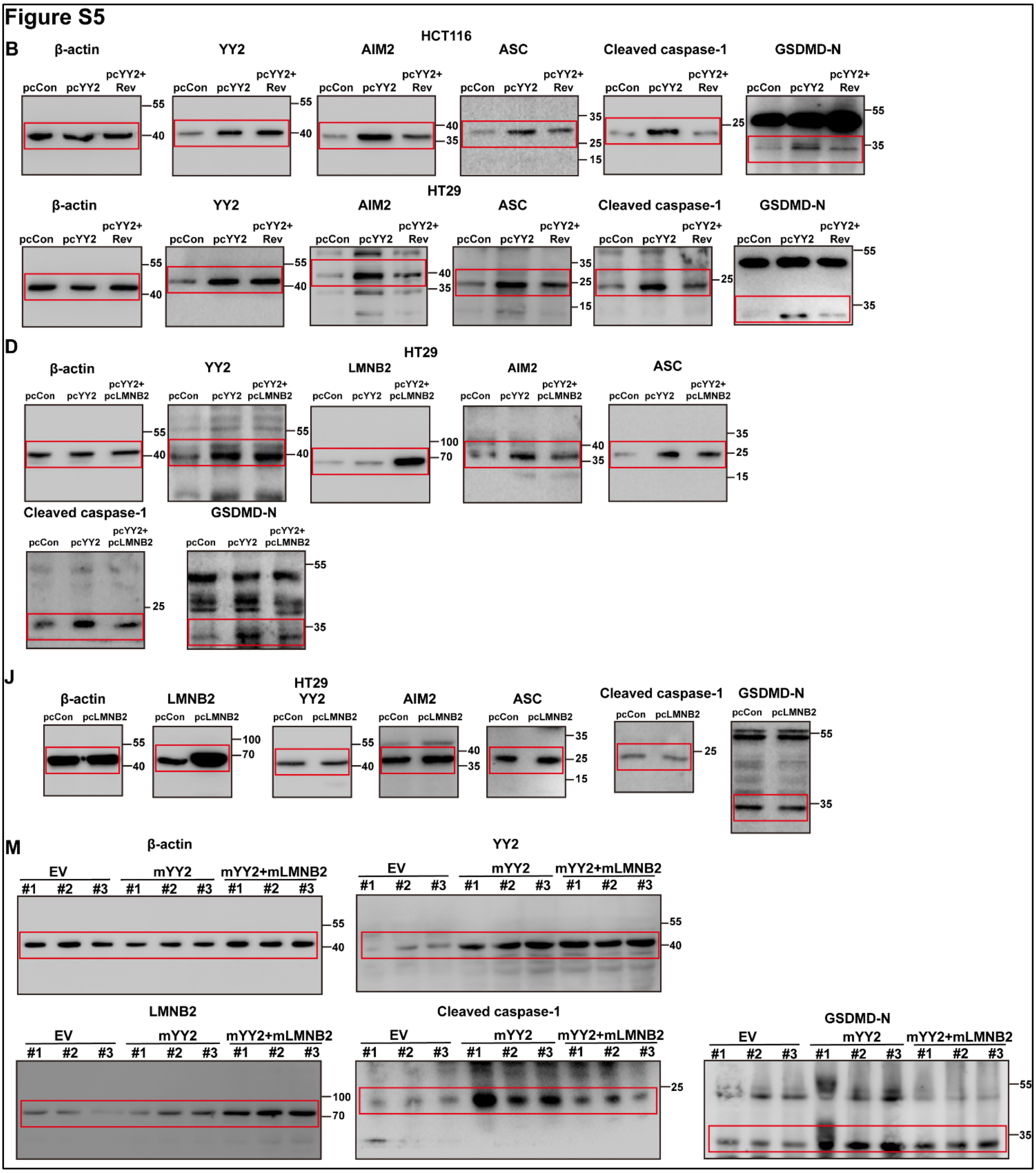

Figure S12. Uncropped western blots with the indicated areas of selection in Figs 4, 5, 6 and Supplementary Figs S1, S2, S4, S5, S7, S8 and S9. (continued)

H

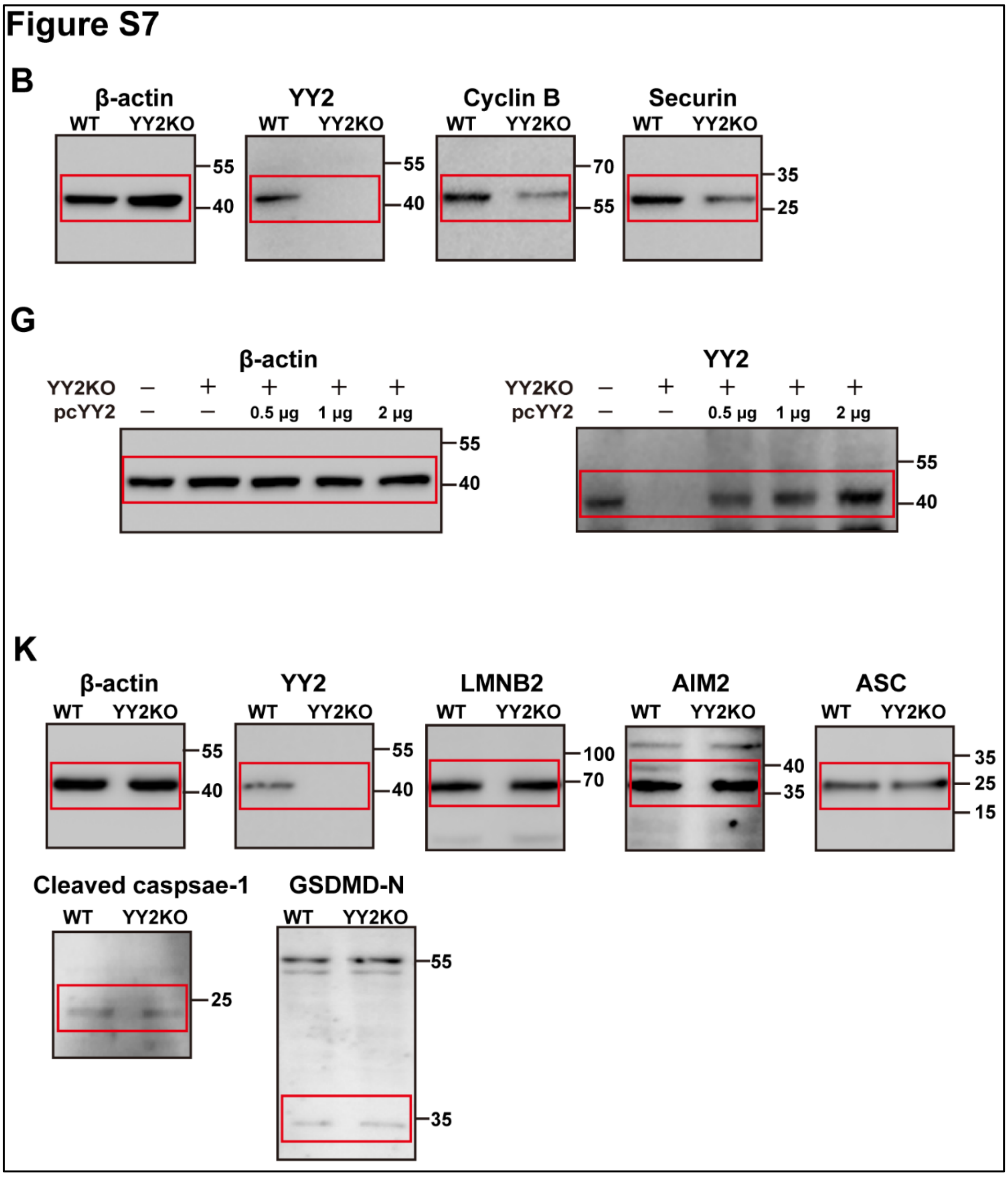

Figure S12. Uncropped western blots with the indicated areas of selection in Figs 4, 5, 6 and Supplementary Figs S1, S2, S4, S5, S7, S8 and S9. (continued)

I

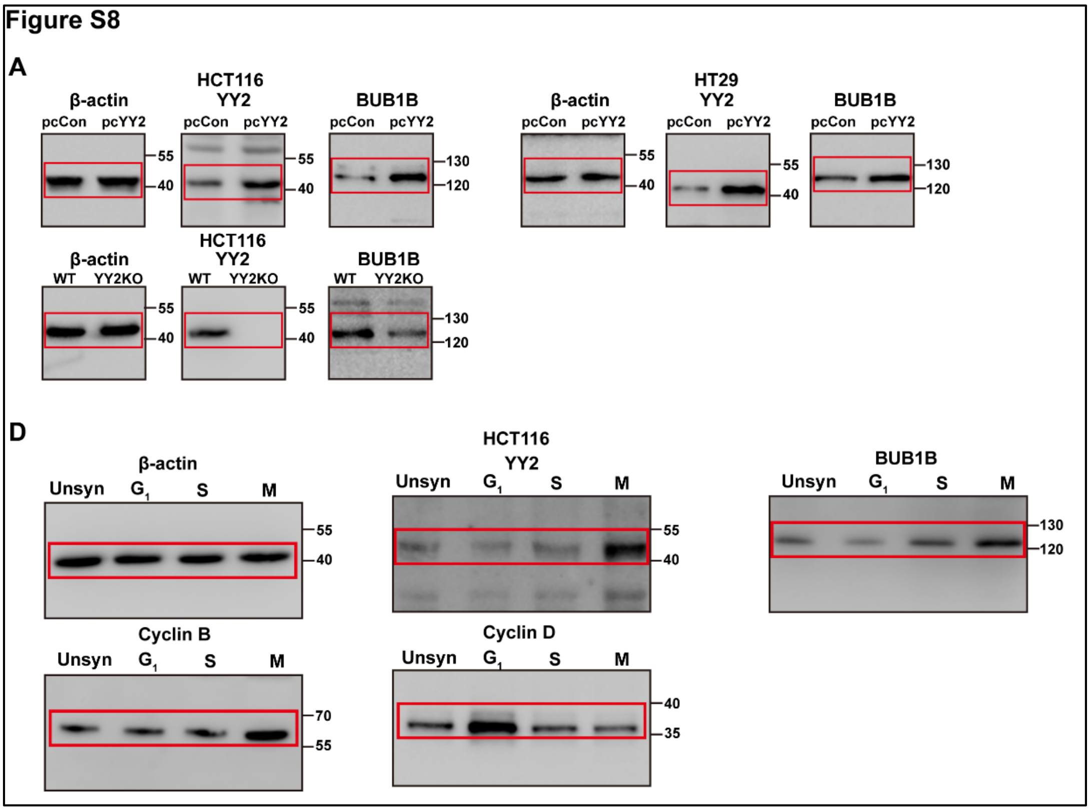

Figure S12. Uncropped western blots with the indicated areas of selection in Figs 4, 5, 6 and Supplementary Figs S1, S2, S4, S5, S7, S8 and S9. (continued)

J

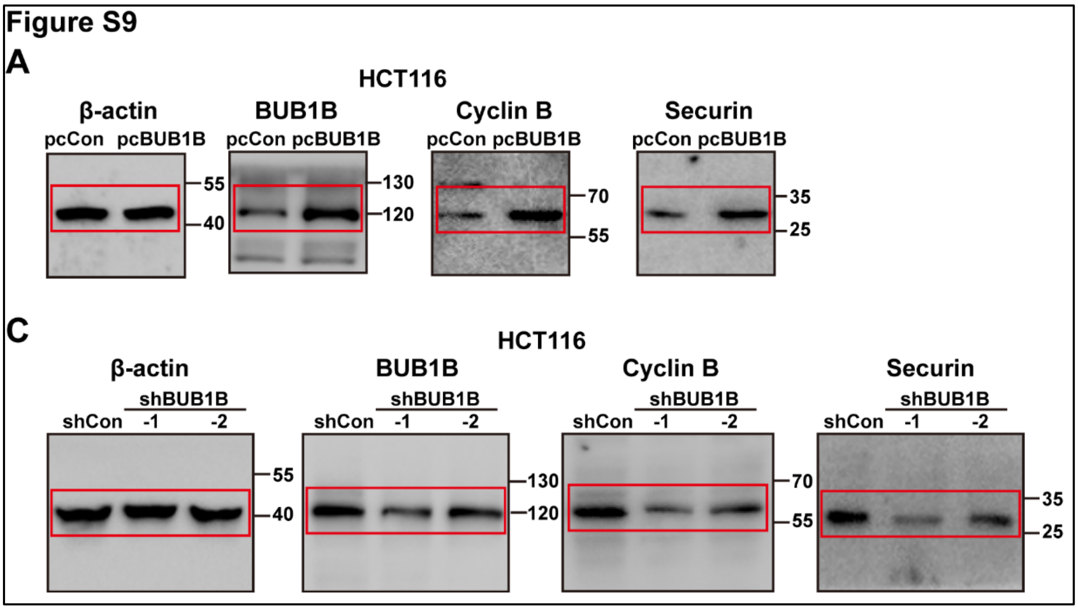

Figure S12. Uncropped western blots with the indicated areas of selection in Figs 4, 5, 6 and Supplementary Figs S1, S2, S4, S5, S7, S8 and S9.

Table S1. Antibodies used for western blotting, ChIP assay, immunohistochemistry, immunofluorescence, flow cytometry, and *in vivo* PD-L1 blockade.

Antibody	Product No.	Maker	Experiment	Dilution
Anti-YY2	sc-374455	Santa Cruz Biotechnology	Western blotting	1/1000
			ChIP<br>Immunohistochemistry	30 μg/mL cell lysate<br>1/100
Anti-cyclin D1	sc-8396	Santa Cruz Biotechnology	Western blotting	1/1,000
Anti-cyclin B1	sc-245	Santa Cruz Biotechnology	Western blotting	1/1,000
Anti-BUB1B	Ab183496	Abcam	Western blotting<br>Immunohistochemistry	1/10,000<br>1/100
Anti-securin	sc-56207	Santa Cruz Biotechnology	Western blotting	1/500
Anti-cleaved-N-GSDMD	Ab215203	Abcam	Western blotting	1/100
Anti-cleaved caspase1	4199T	Cell Signaling Technology	Western blotting	1/1,000
Anti-cleaved caspase4	4450	Cell Signaling Technology	Western blotting	1/1,000
Anti-cleaved caspase5	4429	Cell Signaling Technology	Western blotting	1/1,000
Anti-AIM2	A3356	ABclonal	Western blotting<br>Immunofluorescence	1/1,000<br>1/200
Anti-ASC	ab155970	Abcam	Western blotting	1/10,000
Anti-Lamin B2	Ab151735	Abcam	Western blotting	1/2,000
Anti-β-actin	6009-1-Ig	Proteintech	Western blotting	1/50,000
Goat Anti-Rabbit IgG	ZB2301	ZSGB-BIO	Western blotting	1/10,000
Goat Anti-Mouse IgG	ZB2305	ZSGB-BIO	Western blotting	1/10,000
Anti-Histone H3	17168-1-AP	Proteintech	ChIP	2.5 μg/mL cell lysate
Anti-dsDNA	rDSD/4565	Novus Biologicals	Immunofluorescence	2 μg/ml
in vivo MAb anti-mouse PD-L1	BE0101	BioXCell	*in vivo* PD-L1 blockade	10 mg/kg
FITC-anti-mouse-CD3	17A2	Biolegend	Flow cytometry	1/50
PE-anti-mouse-CD4	GK1.5	Biolegend	Flow cytometry	1/100
APC-anti-mouse CD8α	100711	Biolegend	Flow cytometry	1/100
Percp-cy5.5-anti-mouse-Ki67	16A8	Biolegend	Flow cytometry	1/100
PE-anti-mouse TNFα	MP6-XT22	Biolegend	Flow cytometry	1/100
PE-anti-mouse Tim-3	RMT3-23	Biolegend	Flow cytometry	1/100
FITC-anti-mouse PD-1	135213	Biolegend	Flow cytometry	1/50
FITC-anti-mouse IFN-γ	XMG1.2	Biolegend	Flow cytometry	1/50

Table S2: Primer pairs used for qRT-PCR

Gene	Refseq No.	Forward primer sequence (5'-3')	Reverse primer sequence (5'-3')
YY2	NM_206923.4	GCAGTGGGTGAAGGCCAGGCTG	CGGTGTGGACCAGCTGGTGTCG
BUB1B	NM_002322.6	AACAGAAGGCTGAACCACT	CTCCTACACGGATGATTGG
BIRC5	NM_ 001012270.2	AGGACCACCGCATCTCTACAT	AAGTCTGGCTCGTTCTCAGTG
DNASE1	NM_001351825.2	GGGGACGCAGTAGCCGAGAT	CCACAGGCGGATGGATGAC
DNASE2	NM_001375.3	TCGCCTTCCTGCTCTACAAT	CCCATCTTCGAGAACTGAGC
DNASE3	NM_007248.5	TGCCTTCTGTGTGGATAG	AGTGTAGATGCTGCCTAG
β-actin	NM_001101.3	CGAGCGCGGCTACAGCTT	TCCTTAATGTCACGCACGATTT

Videos Legends

Videos S1 and S2: Time-lapse video of pcCon and pcYY2 HCT116 cells.

Time-lapse video of pcCon (**Video S1**) and *YY2*-overexpressing (**Video S2**) HCT116 cells related to **Figure S1**. The display rate is one frame every 200 millisecond. Still images of this video are shown in **Figure S1M**.

Videos S3 and S4: Time-lapse video of pyroptosis of pcCon and pcYY2 HCT116 cells.

Time-lapse video of pyroptosis of pcCon (**Video S3**) and *YY2*-overexpressing (**Video S4**) HCT116 cells related to **Figure 1**. The display rate is one frame every 200 millisecond. Still images of this video are shown in **Figure 1J**.

Videos S5 and S6: Time-lapse video of wild type and HCT116^{YY2KO} cells.

Time-lapse video of wild type (**Video S5**) and HCT116^{YY2KO} (**Video S6**) cells related to **Figure S7**. The display rate is one frame every 200 millisecond. Still images of this video are shown in **Figure S7A**.

Videos S7 and S8: Time-lapse video of pcCon and pcBUB1B HCT116 cells.

Time-lapse video of pcCon (**Video S7**) and *BUB1B*-overexpressing (**Video S8**) HCT116 cells related to **Figure S9**. The display rate is one frame every 200 millisecond. Still images of this video are shown in **Figure S9B**.

Videos S9 and S10: Time-lapse video of shCon and shBUB1B HCT116 cells.

Time-lapse video of shCon (**Video S9**) and *BUB1B*-knockdown (**Video S10**) HCT116 cells related to **Figure S9**. The display rate is one frame every 200 millisecond. Still images of this video are shown in **Figure S9D**.

Videos S11 to S13: Time-lapse video of pcCon, pcYY2 and pcYY2, shBU1B HCT116 cells.

Time-lapse video pcCon (**Video S11**), *YY2*-overexpressing (**Video S12**), and *BUB1B*-knockdown, *YY2*-overexpressing (**Video S13**) HCT116 cells related to **Figure S9**. The display rate is one frame every 200 millisecond. Still images of this video are shown in **Figure S9G**.
